# Harnessing Dietary Tryptophan: Bridging the Gap Between Neurobiology and Psychiatry in Depression Management

**DOI:** 10.3390/ijms27010465

**Published:** 2026-01-01

**Authors:** Amanda Chabrour Chehadi, Enzo Pereira de Lima, Cláudia Rucco Penteado Detregiachi, Rafael Santos de Argollo Haber, Virgínia Maria Cavallari Strozze Catharin, Lucas Fornari Laurindo, Vitor Engracia Valenti, Cristiano Machado Galhardi, Masaru Tanaka, Sandra Maria Barbalho

**Affiliations:** 1 Department of Biochemistry and Pharmacology, School of Medicine, Universidade de Marília (UNIMAR), Marília 17525-902, SP, Brazilsmbarbalho@gmail.com (S.M.B.); 2 Graduate Program in Structural and Functional Interactions in Rehabilitation, School of Medicine, Universidade de Marília (UNIMAR), Marília 17525-902, SP, Brazil; 3 Division of Cellular Growth, Hemodynamic and Homeostasis Disorders, Graduate Program in Medical Sciences, Faculdade de Medicina, Universidade de São Paulo (USP), São Paulo 01246-903, SP, Brazil; lucasfornarilaurindo@usp.br; 4 Department of Cardiovascular and Metabolic Health, School of Philosophy and Sciences, Universidade Estadual Paulista (UNESP), Marília 17525-900, SP, Brazil; 5 Laboratory for Systematic Investigations of Diseases, Department of Biochemistry and Pharmacology, School of Medicine, Universidade de Marília (UNIMAR), Marília 17525-902, SP, Brazil; 6 Danube Neuroscience Research Laboratory, HUN-REN-SZTE Neuroscience Research Group, Hungarian Research Network, University of Szeged (HUN-REN-SZTE), Tisza Lajos Krt. 113, H-6725 Szeged, Hungary; 7 Department of Biochemistry and Nutrition, School of Food and Technology of Marília (FATEC), Marília 17500-000, SP, Brazil; 8 Department of Research, Research Coordination Center, UNIMAR Charitable Hospital, Universidade de Marília (UNIMAR), Marília 17525-902, SP, Brazil

**Keywords:** major depressive disorder, tryptophan metabolism, kynurenine pathway, phytochemicals, dietary supplements, 5-hydroxytryptophan, gut–brain axis, neuroinflammation, plant extracts, precision nutrition

## Abstract

Major depressive disorder remains a leading cause of disability worldwide, with conventional antidepressants offering incomplete and often transient relief. Mounting evidence highlights disturbances in tryptophan (Trp) metabolism as a key biological axis linking inflammation, neuroplasticity, and mood regulation. Plant-derived compounds that modulate this pathway, including 5-hydroxytryptophan, isoflavones, berberine, and polyphenols, have emerged as promising candidates for integrative treatment strategies. Yet, despite encouraging preclinical and clinical findings, knowledge gaps persist regarding long-term efficacy, mechanistic specificity, and standardized therapeutic protocols. This narrative review explores how Trp modulators influence central and peripheral mechanisms relevant to depression, from serotonergic synthesis and kynurenine shunting to gut–brain–immune interactions. Evidence from animal models and randomized clinical trials is critically synthesized, with particular attention to outcomes on mood stabilization, anxiety reduction, cognitive function, and sleep regulation. Special emphasis is placed on translational potential, methodological limitations, and the need for harmonized research frameworks. Here we highlight that phytochemical interventions represent a mechanistically informed and biocompatible strategy for advancing depression management. By bridging neurobiology and clinical psychiatry, these insights may pave the way for next-generation therapeutics that integrate dietary, microbiota-targeted, and anti-inflammatory approaches. Broader application of this research could ultimately refine personalized psychiatry, expand therapeutic horizons, and contribute to global mental health resilience.

## 1. Introduction

Major depressive disorder (MDD) surpasses most chronic diseases in human toll, ranking consistently among the top causes of years lived with disability [1,2]. More than 300 million people currently experience the condition, and its relapsing course relentlessly erodes well-being, productivity, and social cohesion [2,3]. Annual indirect and direct costs already exceed one trillion United States dollars, weakening national economies while stigma quietly erodes family networks, impairs educational attainment, and narrows life opportunities [4,5]. Conventional monoamine-reuptake-based antidepressants, though invaluable, leave a sizeable fraction of patients with partial or transient relief, highlighting an unmet therapeutic horizon [2]. Recent neurobiological work has illuminated Trp metabolism as a convergent node linking serotonergic tone, KYN-driven neuroinflammation, and gut–brain communication [6,7]. Consequently, nutritional psychiatry is moving to center stage, exploring dietary enrichment, phytochemical extracts, and complex plant polysaccharides that can steer Trp flux toward antidepressant pathways [3,7]. These multitarget, biocompatible candidates warrant rapid, translational exploration to transform MDD management [3,8].

5-HT occupies the fulcrum of affective neurocircuitry, yet its synthesis depends on a single dietary precursor, Trp [9,10]. When circulating 5-HT dwindles—whether through genetic polymorphisms, persistent inflammation, or inadequate intake—clinical and pre-clinical data reveal cascades of anhedonia, low mood, and suicidality [9,10]. Supplementation with Trp and, more recently, nanozyme-accelerated biosynthesis swiftly restores synaptic stores, underscoring causality [11,12]. Because no alternative substrate can replenish this indoleamine, strategies that enrich Trp availability or steer its fate toward the serotonergic branch merit priority; they simultaneously quell microglial inflammation and normalize behavior in rodents and humans [10,12]. The problem deepens when pro-inflammatory cytokines activate indoleamine 2,3-dioxygenases (IDOs), shunting Trp down the KYN track, exhausting 5-HT, and generating neurotoxic QA [10,13]. Meta-analytic biomarker work links this detour to melancholic features, treatment resistance, and suicidal intent, framing KYN dysregulation as a mechanistic bottleneck [9,10]. Encouragingly, plant-derived agents—ginsenoside Rk3, *Pulsatilla* saponins, *Hypericum* extracts, diverse polysaccharides, and indole alkaloids—re-calibrate this junction [12,14,15]. By activating tryptophan hydroxylase (TPH), repressing IDO1, re-shaping gut microbiota, and enriching anti-inflammatory indoles, they restore serotonergic dominance and offer a multimodal, biocompatible blueprint for next-generation antidepressant therapy [10,14].

**Figure 1:**
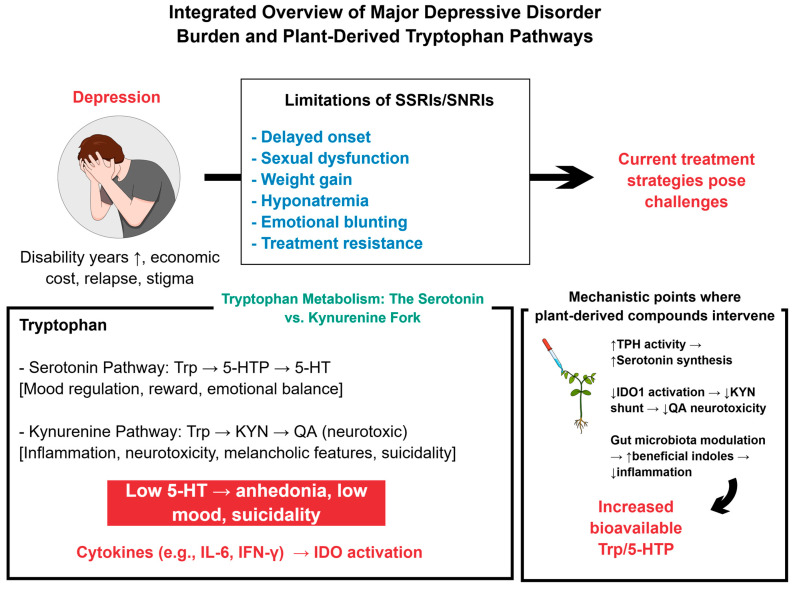
Integrated overview of major depressive disorder burden and plant-derived modulation of tryptophan pathways. This schematic summarizes the clinical burden of major depressive disorder (MDD) and highlights limitations of current selective serotonin (5-HT) reuptake inhibitor (SSRI) and 5-HT-norepinephrine reuptake inhibitor (SNRI) therapies, including delayed onset, sexual dysfunction, weight gain, hyponatremia, emotional blunting, and treatment resistance. Tryptophan metabolism is illustrated as a fork between the 5-HT pathway (promoting mood regulation and emotional balance) and the kynurenine (KYN) pathway (associated with inflammation, neurotoxicity, and melancholic features). Inflammatory cytokines such as interleukin (IL)-6 and interferon (IFN)-γ shift tryptophan away from 5-HT production by activating Indoleamine 2,3-dioxygenase (IDO), contributing to reduced 5-HT, anhedonia, low mood, and suicidality. Mechanistic points where plant-derived compounds may intervene are shown on the right, including enhancement of tryptophan hydroxylase (TPH) activity and 5-HT synthesis, inhibition of IDO1 activation to reduce neurotoxic KYN metabolites, and modulation of the gut microbiota to increase beneficial indoles and reduce inflammation. Collectively, these actions increase bioavailable tryptophan and 5-HTP, offering potential complementary strategies for improving depressive symptoms. Created using Mind the Graph (https://mindthegraph.com/, last accessed on 6 December 2025). Abbreviations: Trp, Tryptophan; 5-HTP, 5-Hydroxytryptophan; KYN, Kynurenine; QA, Quinolinic acid.

Selective 5-HT and 5-HT-norepinephrine reuptake inhibitors still headline pharmacological guidelines for MDD, yet their benefit remains modest [16,17]. Clinical relief often arrives only after a fortnight—a critical delay for individuals experiencing suicidal ideation or severe impairment [16,18]. Adverse events accumulate in the interim: sexual dysfunction that may persist beyond discontinuation; progressive weight gain and hyponatremia threaten metabolic and electrolyte balance; emotional blunting, apathy, and cognitive fog sap motivation and adherence, especially in adolescents and older adults vulnerable to withdrawal syndromes [16,18,19,20]. Some patients become treatment-resistant after adequate trials, and relapse still strikes a part of the apparent responders within a year, each episode deepening psychosocial impairment and economic burden [18,19,21]. These intertwined shortcomings have energized the search for nutraceutical, phytochemical, and mind–body interventions [18,22,23,24]. Omega-3 fatty acids, flavonoid-rich extracts, acupuncture, mindfulness practice, and guided psilocybin sessions already display encouraging efficacy with fewer liabilities, earning cautious endorsement in emerging guidelines and stimulating expansive translational research [22,23,24,25]. Together, these trends justify systematic appraisal of plant-derived Trp as the next therapeutic frontier [18,22,26]. Figure 1 illustrates an overview of MDD burden and plant-derived modulation of Trp pathways.

Plant-derived modulators of Trp metabolism have moved from folklore to the laboratory, carrying a mechanistic sophistication once reserved for synthetics [27,28,29]. Oligosaccharides from *Morinda officinalis* boost gut 5-hydroxy-L-tryptophan, elevating brain 5-HT and reversing stress-induced anhedonia [14,27,28]. Ginsenoside Rk3 and *Pulsatilla* saponins suppress IDOs, redirect KYN flux, and calm neuroinflammation, while *Hypericum* fractions, probiotic indoles, and alkaloid derivatives further polish gut–brain signaling with striking behavioral gains [28,29,30]. These findings echo an ethnopharmacological legacy: lavender, turmeric, bacopa, the Chinese formula Xiaoyao-san, and *Ayurvedic rasayanas* have eased melancholy for centuries, with numerous mood-modulating plants catalogued across ancient pharmacopoeias [27,28,30]. Their renaissance coincides with nutritional psychiatry, a field treating diet as neuronal software by integrating microbiome science, polyphenol chemistry, and amino-acid neuroscience [14,27,28]. Some global guidelines now acknowledge nutraceuticals and phytoceuticals, and bibliometric analyses chart exponential growth in phytotherapeutic trials [27,28,29]. Moreover, xylopic acid, sclareol-quercetin blends, capsaicin, and Chaihu-Shugan-San enhance SSRI efficacy while tempering metabolic and cognitive liabilities, underscoring the promise of safer, synergistic, multicomponent antidepressant strategies [27,28,29].

The primary objective of this review is to systematically examine the efficacy and neurobiological mechanisms of plant-derived Trp in the treatment of depression. Because plant-derived Trp compounds act across multiple interacting pathways, a comprehensive synthesis requires revisiting core mechanisms, such as TPH activity, KYN metabolism, cytokine signaling, and gut microbiota function, since each reappears in distinct clinical and translational contexts. By addressing both experimental and clinical evidence, the review seeks to clarify how interventions targeting Trp metabolism may influence mood regulation and therapeutic outcomes [31,32,33]. A central aim is to bridge the gap between fundamental neurobiology and clinical psychiatry, offering an integrated perspective that connects molecular mechanisms with patient-centered applications. Specifically, the review focuses on delineating the pathways through which Trp and its metabolites modulate the serotonergic and KYN systems, evaluating preclinical and clinical trial data, and identifying limitations that must be overcome to enhance translational relevance. Attention is given to unresolved issues in bioavailability, pharmacokinetics, and long-term safety. Ultimately, this review highlights future therapeutic prospects and emphasizes the importance of integrating mechanistic insights with clinical practice to guide precision-based strategies for depression management. 

In contrast to prior reviews that mainly describe generic nutritional strategies or pharmacological manipulation of Trp pathways, our focus is on plant derived sources and modulators of Trp metabolism as a distinct therapeutic domain. Plant derived Trp reaches the brain embedded in complex food matrices that contain fibers, polyphenols, alkaloids, and saponins, which jointly reshape intestinal absorption, microbiota composition, and cytokine driven regulation of key enzymes such as IDO, tryptophan 2,3-dioxygenase (TDO), and kynurenine 3-monooxygenase (KMO). Non-plant derived tryptophan sources, including animal proteins and purified supplements, primarily increase systemic substrate availability but provide little direct modulation of these upstream regulatory nodes. By explicitly contrasting these metabolic routes, the review proposes that plant-derived Trp is not only a precursor for 5-HT but also a systems level regulator of peripheral and central Trp flux, offering a mechanistically distinct and potentially safer pathway to rebalance the 5-HT, kynurenine (KYN), and indole branches in depression.

This review carries significant implications for psychiatry, nutritional sciences, and integrative medicine by advancing understanding of plant-derived Trp as a therapeutic option for depression. Its contributions extend beyond cataloguing current evidence, offering a critical synthesis that highlights both mechanistic insights and clinical applications. By evaluating how dietary and phytochemical interventions influence Trp metabolism and related neurobiological pathways, the review provides an innovative framework with direct translational relevance. The discussion emphasizes how such approaches may complement existing pharmacological treatments, reduce the burden of side effects, and support individualized, nutrition-based strategies in mental health care. Notably, the review underscores the potential of integrating dietary science with neuropsychiatric research to broaden treatment paradigms and improve patient outcomes. Here, we highlight the necessity of sustained interdisciplinary collaboration, uniting psychiatry, molecular biology, nutrition, and clinical practice. Only through such efforts can the full therapeutic potential of Trp-related interventions be realized in depression management.

## 2. Neurobiology of Depression: Beyond Serotonin (5-HT)

For much of the twentieth century, textbooks portrayed depression as a straightforward consequence of diminished synaptic 5-HT, a view seemingly vindicated by the success of selective serotonin reuptake inhibitor (SSRIs) and reinforced by simplified biochemical schematics [34,35,36]. Accumulating evidence now unpicks that narrative. An umbrella analysis fails to locate a reliable serotonergic deficit signature in MDD. At the same time, parallel studies reveal stress-evoked glutamatergic drift at AMPA receptors, a shift that disrupts excitatory balance and long-term potentiation [34,37,38,39]. Cytokine surges further interweave immune and neural realms, diverting Trp toward KYN metabolites with neurotoxic or neuroprotective potential, and tuning mitochondrial resilience in vulnerable circuits [40,41,42]. Animal paradigms of chronic unpredictable stress echo these molecular fingerprints, exposing dendritic retraction and synaptic pruning that compromise mood-regulating networks [34,41,42]. Against this backdrop, neuroplasticity models integrate receptor interactions, neurotrophic signaling, and gliovascular dynamics, offering a more comprehensive representation of the disorder’s heterogeneity [34,35,40]. Such multi-layered models do more than widen mechanistic horizons; they steer translational efforts toward modulators of plasticity, inflammation, and metabolism, promising interventions that transcend the monoaminergic lens and address the complex biology underlying depressive illness [40,43,44,45].

### 2.1. Microglia Activation and Neuroinflammation

Microglia constantly monitor the brain. They prune synapses and release trophic factors that help maintain metabolic and neural networks [46,47,48]. Stress shifts them into a reactive mode. In this state, they proliferate more rapidly, alter their metabolism, and release inflammatory signals that weaken excitatory synapses [46,47,49]. Glucocorticoids and catecholamines coursing through HPA and sympathetic pathways reinforce this shift, entrenching microglia in maladaptive, inflammatory phenotypes [46,50,51,52]. A spectrum of reactive states emerges: some microglia secrete cytokines and reactive metabolites that fracture neuronal integrity, while others attempt repair through anti-inflammatory mediators [48,53,54]. Early-life immune challenges further dysregulate microglial surveillance, triggering excessive dendritic spine engulfment and predisposing adolescents to mood disturbances [55,56,57]. In adulthood, sustained IL-6 release drives hippocampal astrocyte apoptosis, and prostaglandin signaling in striatal regions amplifies negative affect [49,58,59]. Crucially, pharmacological or genetic dampening of microglial activation reduces cytokine burden and restores synaptic function, alleviating depressive-like behaviors in animal models [48,49,58]. Such findings underscore microglia’s dual capacity for neuroprotection and neurotoxicity, as well as highlight inflammatory modulation as a promising axis for next-generation antidepressant development [46,48,49].

A consistent pattern emerges across studies. IL-1β, IL-6, TNF-α, and IFN-γ tend to rise with symptom severity and often predict relapse. Stress increases these cytokines via NLRP3 activation, while epigenetic regulators such as EZH2 further amplify them. Together, they build an inflammatory loop that shapes mood and cognition [60,61,62]. Meta-analytic syntheses, large observational cohorts, and translational models consistently show that concentrations of these cytokines rise in parallel with symptom load, forecast relapse, and differentiate treatment-resistant cases from treatment-responsive patients [63,64,65]. Stress priming activates NLRP3 inflammasomes, unleashing bursts of IL-1β that propagate glial crosstalk, while epigenetic regulators such as EZH2 intensify IL-6 and TNF-α transcription, accelerating synaptic attrition and anhedonia [66,67,68]. Longitudinal work in adolescents demonstrates that elevated baseline TNF-α or IL-6 anticipates chronic anhedonia and white-matter dysconnectivity years later, underscoring a trajectory of immune-driven neuroprogression [69,70,71]. Complementary primate and lipopolysaccharide paradigms replicate the cytokine surge and its behavioral sequelae, bolstering causality [72,73,74]. Crucially, immunomodulatory interventions can reverse this profile: ketamine lowers IL-6 and TNF-α in treatment-resistant depression in tandem with rapid affective recovery, and targeted anti-cytokine therapies normalize peripheral markers while lifting mood in case studies and pilot trials [75,76,77]. Together, these findings portray a feed-forward inflammatory circuit that not only marks depressive severity and chronicity but also delineates mechanistic targets for next-generation precision antidepressants, with potential for biomarker-guided stratification in clinical practice worldwide [72,73,78]. These inflammatory shifts directly reshape the neurogenic niche, setting the stage for changes in hippocampal plasticity.

### 2.2. Neurogenesis Impairment and Hippocampal Atrophy in Depressive Pathology

The hippocampus continually produces new granule cells. These cells integrate into memory and emotion circuits, helping maintain cognitive flexibility and emotional stability. Rodent, primate, and human proxy experiments reveal that boosting this renewal before stress inoculates against anhedonia and cognitive drift, whereas blocking it magnifies vulnerability and depressive phenotypes [79,80,81]. Mitochondrial fitness, autophagic clearance, and neuroimmune balance calibrate neurogenic yield, linking cellular housekeeping to psychological health and nominating the neurogenic niche as a tractable antidepressant target [82,83,84]. Chronic stress destabilizes this balance. Elevated corticosterone primes microglia and activates NF-κB. As a result, IL-1β, IL-6, TNF-α, and IFN-γ accumulate in the hippocampus. Because microglia are the primary source of many of these cytokines, their activation acts as a bottleneck for neurogenesis, constraining proliferation and survival of newborn neurons. These cytokines stop progenitors from dividing. They also divert BDNF signaling through CDK5-phosphorylated huntingtin. At the same time, astrocytes provide less VEGF, and neural precursors are pushed toward apoptosis [85,86,87]. Glial activation amplifies oxidative damage, ADAM17/TNF-α signaling, and aberrant pruning, cementing a neurogenic standstill that melatonin or ginsenoside Rg1 can dismantle [88,89,90]. Structural imaging corroborates the cellular narrative: meta-analyses and cross-diagnostic cohorts consistently report bilateral, often left-dominant, hippocampal atrophy in depression [87,90,91]. Seven-Tesla datasets pinpoint shrinkage to dentate gyrus and tail subfields, especially in treatment-resistant illness [89,91,92]. Interventions that rekindle plasticity, including electroconvulsive therapy and aerobic exercise, partially restore volume, implying that suppressed neurogenesis and dendritic retraction are reversible contributors to mood recovery [89,93,94]. Such structural reversibility converges with behavioral improvements, reinforcing adult neurogenesis as both a biomarker and a mediator of durable resilience [95,96].

Chronic neuroinflammation progressively dismantles the hippocampal neurogenic niche. Activated microglia, astrocytes, and infiltrating immune cells inundate progenitor zones with IL-1β, caspases, reactive oxygen species, and other cytotoxic mediators, stalling cell-cycle progression and truncating dendritic maturation [97,98,99]. Sustained IL-10 exposure or traumatic injury reprograms microglia toward a phagocytic, synapse-stripping phenotype that disrupts neuron–glia crosstalk and shortens the lifespan of newborn neurons [100,101,102]. Intrinsic genetic vulnerabilities intensify the problem: loss of the transcription factor Tcf4 unleashes latent inflammatory pathways inside neural stem cells, extinguishing their regenerative capacity [97,98]. Systemic infections or chronic gut inflammation widen this suppression, propagating cytokine waves that reverberate into cognitive decline and depressive affect [103,104]. Compounding these injuries, elevations in IL-6 and TNF-α draw down BDNF, a molecule essential for synaptic refinement and mood stability, thereby tightening the grip of inflammation on neuroplasticity [105,106,107]. Clinical imaging and behavioral studies mirror these molecular events: higher cytokine loads predict smaller hippocampi, poorer memory, and deeper anhedonia, while agents that either quiet neuroimmune signaling or re-energize neurogenesis—such as *Akebia* saponin D, liraglutide, naringenin, or KYN-pathway modulators—restore plasticity and relieve symptoms [105,108,109,110,111].

### 2.3. The Link Between Inflammation, Tryptophan (Trp) Metabolism, and Depressive Symptoms

**Figure 2:**
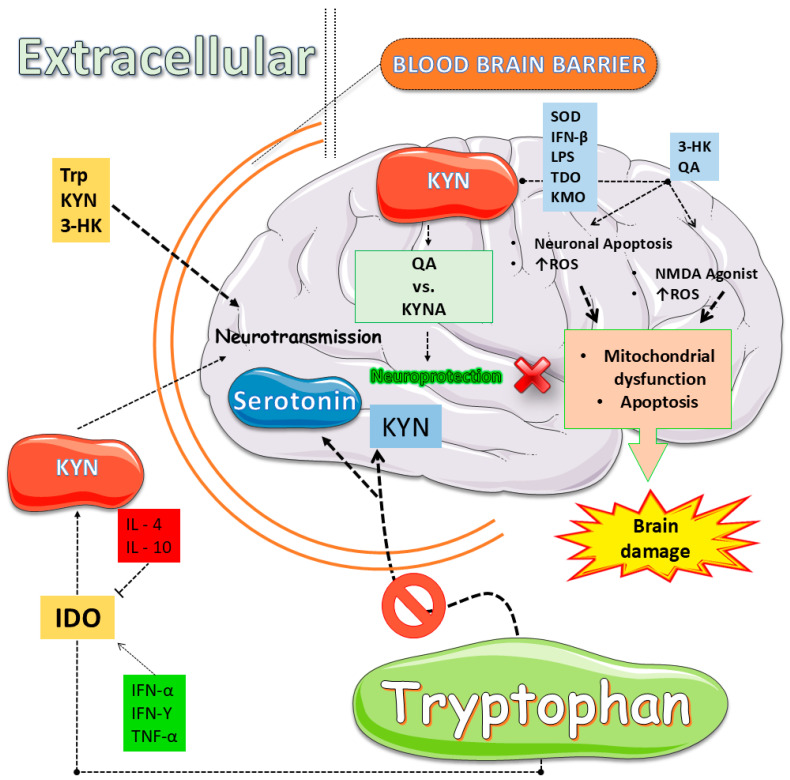
Tryptophan (Trp) metabolism through the kynurenine (KYN) pathway: balancing neuroprotection and neurotoxicity in depression. This figure illustrates how immune-mediated activation of the KYN pathway disrupts Trp metabolism, linking inflammation with depression-related neuropathology. The stress hormone cortisol stimulates tryptophan 2,3-dioxygenase (TDO). Pro-inflammatory cytokines [interferon (IFN)-alpha (α), IFN-beta (β), IFN-gamma (γ), tumor necrosis factor alpha (TNF-α)] and lipopolysaccharide (LPS) induce indoleamine 2,3-dioxygenases (IDOs), diverting Trp away from serotonin (5-HT) synthesis toward KYN production. In contrast, anti-inflammatory cytokines [interleukin (IL)-4, IL-10] and superoxide dismutase (SOD) counteract this process. Oxygen molecule (O_2_), IFN-β, IFN-γ, and TNF-α stimulate and IL-4, IL-10, and SOD suppress kynurenine 3-monooxygenase (KMO). Furthermore, IDOs, TDO, and KMO activities are influenced by positive and negative feedback loops. Peripheral KYN crosses the blood–brain barrier, where it undergoes two competing fates: (i) conversion into kynurenic acid (KYNA), a putative neuroprotective N-Methyl-D-Aspartate (NMDA) receptor antagonist with putative antioxidant properties, or (ii) metabolism into 3-hydroxykynurenine (3-HK) and quinolinic acid (QA), which promotes reactive oxygen species (ROS), NMDA receptor overstimulation, mitochondrial dysfunction, and neuronal apoptosis, ultimately leading to neurodegeneration. The dashed inhibitory arrow indicates that enhanced KYN metabolism reduces 5-HT and melatonin synthesis, impairing neurotransmission and neuroprotection. The balance between KYNA and QA determines whether the KYN pathway promotes resilience or vulnerability to depression. Abbreviations: ↑, Increase.

Innate and adaptive immune signals divert Trp away from serotonergic fate, engaging IDOs and TDO that accelerate the KYN cascade of neuroactive compounds [112,113]. IFN-γ, IL-6, and TNF-α boost these enzymes across the gut and brain, shrinking 5-HT stores while raising KYN and its downstream quinolinate products [113,114]. Chronic stress or intestinal inflammation magnifies the shift, elevating KYN-to-5-HT ratios in rodents and paralleling anhedonia in patients [113,115]. This metabolic rerouting, therefore, links immune activation to depressed mood and cognitive decline [113,115,116,117] (Figure 2). Yet the pathway’s end stage proves most damaging. QA overstimulates NMDA receptors, drives calcium overload, and sparks oxidative stress [113,114,118,119,120]. Traumatic brain injury or systemic inflammation upregulates KYN monooxygenase, intensifying quinolinate build-up, halting progenitor proliferation, and eroding hippocampal circuits [112,113,118]. Microplastic exposure yields similar DNA fragmentation and microglial activation [112,113,121]. Therapeutic blockade with ketamine or selective enzyme inhibitors reduces quinolinate levels, restores neurogenesis, and rescues behavior [112,113,114]. Reviews across neurodegenerative contexts confirm that balancing toxic and protective KYNs is critical for hippocampal integrity and mood regulation [112,113,115,122]. Collectively, these findings reposition the KYN axis as both a biomarker and a mechanistic driver, inviting precision anti-inflammatory or metabolic interventions that could recalibrate Trp flux, preserve plasticity, and alleviate depressive illness.

Chronic immune activation diverts Trp metabolism by upregulating IDOs and TDO, drawing substrate from 5-HT synthesis and inflating production of neuroactive KYNs such as QA [113,123,124]. Quinolinic excess overstimulates NMDA receptors, kindles oxidative stress, and arrests hippocampal neurogenesis, thereby reducing BDNF, pruning synapses, and disturbing gut–brain communication essential for mood and memory [123,125]. These pathophysiological threads recast depression as a metabolic-immune-plasticity disorder that demands therapies surpassing monoaminergic repair [113,123,124]. Evidence now endorses blended strategies that curb inflammation, recalibrate microbiota signaling, temper glutamatergic drive, and reignite neurogenesis [126,127]. Phytochemicals illustrate this integrative ethos: polyphenols, soy isoflavones, berberine, albiflorin, *Pulsatilla* saponins, and naringenin jointly silence microglial cytokine release, inhibit IDO1, activate tryptophan hydroxylase, and foster survival of newborn neurons, collectively improving affective and cognitive outcomes in stress-resistant models [128,129,130]. Such multi-layered modulation repairs molecular circuitry while offering a blueprint for precision nutrition and pharmacology that mirrors depression’s multifactorial biology, promising durable relief in clinical practice [131,132,133].

## 3. Tryptophan (Trp) Metabolism: Central Pathways and Peripheral Influences

This review uses the term plant-derived Trp compounds to refer to plant Trp sources, purified plant Trp-based supplements, and phytochemicals that modulate Trp metabolism. Shortened forms such as “Trp” or “phytochemicals” are used only when the context is specific. For clarity, non-plant derived Trp in this review refers to endogenous pools generated by protein turnover, dietary intake from animal products, and synthetic formulations of L-tryptophan or 5-hydroxytryptophan that are administered as isolated nutrients or drugs. Once absorbed, these sources largely share the canonical metabolic fates of Trp through the 5-HT, KYN, niacin, and indole pathways, and their central effects mainly depend on competition with other large neutral amino acids at the blood brain barrier and on inflammatory control of IDO and TDO activity. Their impact is therefore dominated by shifts in systemic substrate availability and by interactions with pharmacological agents such as antidepressants or cytokine modulators.

Plant derived Trp metabolism differs in three interlinked ways. First, plant proteins and extracts deliver Trp together with high amounts of fibers, resistant starches, and polyphenols that slow intestinal transit, alter luminal pH, and favor microbial species that convert Trp into indoles with antioxidant and aryl hydrocarbon receptor activity. Second, many phytochemicals, including isoflavones, berberine, and flavonoids, act directly on Trp pathway enzymes by downregulating IDO1 or KMO and by supporting TPH activity, which shifts flux away from neurotoxic kynurenines toward serotonin and protective KYNA. Third, plant matrices and their metabolites act on immune and endocrine signaling, lowering pro-inflammatory cytokines that otherwise drive pathological KYN production. Together, these properties give plant-derived Trp a unique capacity to influence both the quantity of available substrate and the qualitative pattern of downstream metabolites, in contrast to non-plant derived sources that mostly affect the former.

Trp metabolism lies at the heart of neuropsychiatric research, serving as a biochemical crossroad between neurotransmission, immune regulation, and circadian control [134,135]. Clinical depression emerges from disruptions in these same pathways because even subtle shifts in 5-HT or KYN flux can alter neuroplasticity, inflammatory tone, and stress responsiveness. Among its metabolic fates, the 5-HT and KYN pathways represent fundamental routes with profound implications for mood and cognition [136,137]. The 5-HT branch underpins neurotransmitter synthesis and neuroplasticity, while the KYN branch generates a spectrum of neuroactive metabolites that shape neuroinflammation and excitotoxicity [138,139]. Dysregulation of this delicate balance has been repeatedly implicated in depression, schizophrenia, and neurodegenerative disorders [13,140]. This section introduces these dual metabolic routes, emphasizing their molecular complexity, clinical relevance, and potential as therapeutic targets for precision psychiatry.

### 3.1. Serotonin (5-HT) and Kynurenine (KYN) Metabolic Pathways: Fundamental Metabolic Routes

**Figure 3:**
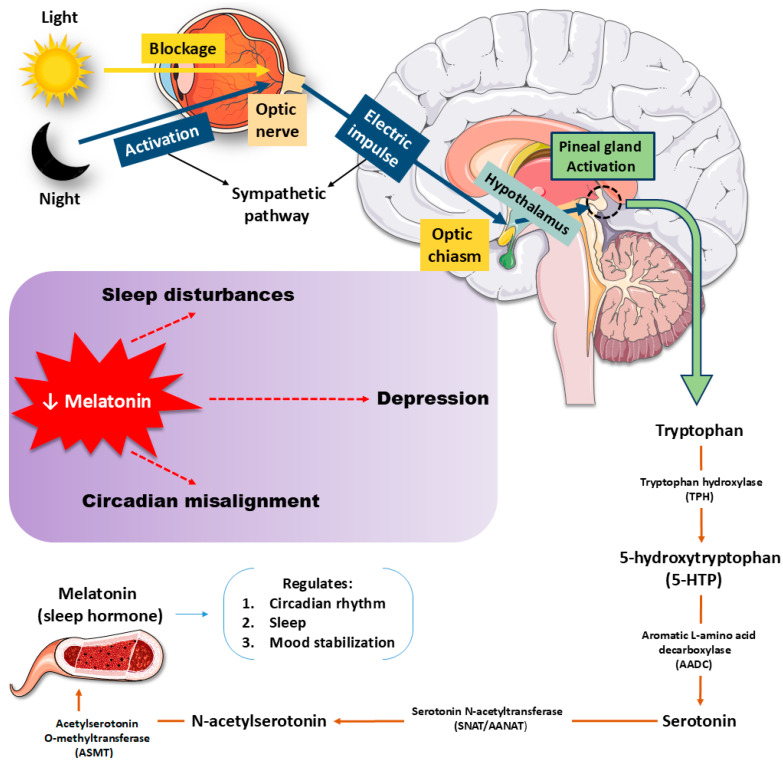
The light–suprachiasmatic nucleus (SCN)–pineal pathway regulating melatonin biosynthesis. Light information from the retina reaches the suprachiasmatic nucleus (SCN), the master circadian pacemaker, via the retinohypothalamic tract. The SCN projects to the paraventricular nucleus and modulates sympathetic innervation of the pineal gland. In darkness, SCN activity stimulates pineal metabolism of Trp through a sequential enzymatic cascade: tryptophan hydroxylase (TPH) converts Trp into 5-hydroxytryptophan (5-HTP), aromatic L-amino acid decarboxylase (AADC) generates serotonin (5-HT), serotonin N-acetyltransferase (SNAT) produces N-acetylserotonin, and acetylserotonin O-methyltransferase (ASMT) catalyzes the final step to melatonin. Light exposure suppresses this pathway, reducing melatonin output. Melatonin acts as a key circadian regulator, influencing sleep, neuroendocrine rhythms, and mood regulation. Abbreviations: ↓, Decrease.

The 5-HT pathway begins when Trp is hydroxylated by TPH1 and TPH2, producing 5-HTP, which is then decarboxylated to form 5-HT [141,142]. This neurotransmitter engages multiple receptor families, including 5-HT1A, 5-HT2A, 5-HT4, and 5-HT6, each of which exerts distinct influences on mood regulation, neurogenesis, and synaptic plasticity [134,141]. Antidepressants such as SSRIs not only increase synaptic 5-HT but also enhance structural remodeling and circuit flexibility, shifting the focus from a deficit model to a neuroplasticity framework [142,143]. Receptor–receptor interactions, like 5-HT2A–TrkB heterodimerization, and receptor-specific mechanisms, such as biased 5-HT1A signaling or 5-HT4-mediated memory modulation, highlight the pathway’s molecular sophistication [13,143]. Psychedelic compounds further reveal how 5-HT2A activation may promote cortical plasticity, though 5-HT itself may not be the primary ligand driving such effects [134,142]. Yet, pressing gaps persist. The precise endogenous mechanisms underlying receptor-driven plasticity remain unclear, while phase-specific roles of 5-HT in declarative memory are insufficiently mapped. Translational barriers hinder the movement of receptor-targeted and psychedelic-based therapies into clinical use, particularly regarding long-term safety. Moreover, the interplay of diet, stress, and genetic variability complicates predictions of therapeutic response [134,143]. Clarifying these complexities is critical for developing precise, individualized treatments for mood and cognitive disorders. Figure 3 illustrates the multiple biological effects associated with melatonin.

The KYN metabolic pathway represents the primary route of Trp degradation, initiated by the rate-limiting enzymes IDOs and TDO, leading to the production of KYN [144,145]. This intermediate can be further metabolized into neuroactive derivatives with contrasting effects: KYNA, an NMDA receptor antagonist with neuroprotective properties, and QA, an NMDA receptor agonist with excitotoxic and pro-inflammatory actions [146,147]. Additional metabolites such as 3-HK contribute to oxidative stress, linking the pathway to neurodegenerative and psychiatric disorders [144,146]. Meta-analyses and experimental studies consistently show a pathological shift toward neurotoxic metabolites in depression, schizophrenia, Alzheimer’s disease, and neurodegeneration [13,148]. Yet significant challenges persist. The dichotomy of KYNA as purely protective and QA as strictly harmful is now recognized as overly simplistic since their effects vary with concentration, brain region, and disease context [149,150]. Furthermore, the spatial distribution and regulation of key enzymes such as KMO, KATs, and QPRT remain insufficiently characterized in both central and peripheral systems [151,152]. Peripheral metabolite measures often fail to accurately represent brain activity, limiting their use as biomarkers [152,153]. Finally, although enzyme inhibitors and analogues are under clinical investigation, their long-term efficacy and safety are far from established [151,154].

### 3.2. Oxidative Stress and Inflammation: Drivers of Kynurenine (KYN) Pathway Activation

Trp is an essential amino acid indispensable for neurochemical homeostasis, mood regulation, and cognitive integrity [13,123,135]. While a minor fraction enters the 5-HT pathway to support neurotransmission and neuroplasticity, the vast majority is shunted into the KYN pathway, generating metabolites with neuroactive and immunomodulatory properties [10,113,135]. Cytokine-driven diversion of Trp toward the KYN branch, described earlier, amplifies oxidative stress and metabolic imbalance, a shift consistently observed in depression, schizophrenia, and neurodegenerative disorders [13,113,155]. Accumulation of neurotoxic metabolites, such as QA, exacerbates excitotoxicity and oxidative stress, whereas KYNA may provide neuroprotection in a context-dependent manner [156,157,158]. Despite clear links between immune activation, oxidative stress, and altered Trp metabolism, significant questions remain unresolved [13,113,156]. The molecular crosstalk among cytokine signaling, mitochondrial dysfunction, and metabolic reprogramming remains unclear [13,156,159]. Similarly, the role of gut microbiota and platelets as peripheral modulators of Trp fate is incompletely defined [109,115,136,160]. Translational gaps persist in developing reliable biomarkers and effective therapeutic strategies, particularly antioxidants, pathway modulators, and microbiota-targeted interventions [13,156,159]. Addressing these challenges will be crucial for precision approaches to neuropsychiatric and neurodegenerative diseases.

IDOs and TDO catalyze the first, rate-limiting step of the KYN pathway, thereby functioning as pivotal gatekeepers of Trp metabolism [161,162,163]. Under conditions of inflammation and oxidative stress, these enzymes are strongly upregulated: TDO, primarily expressed in the liver and regulated by systemic Trp levels and glucocorticoids, and IDO, widely induced in immune cells by cytokines such as IFN-γ and TNF-α [162,164,165]. Their activation diverts Trp away from 5-HT synthesis and channels it toward KYN, which, in turn, generates metabolites such as QA, with excitotoxic and pro-inflammatory effects, and KYNA, with context-dependent neuroprotection [162,163,164]. Beyond enzymatic activity, KYN itself acts as an AhR ligand, modulating immune tolerance and tumor progression [161,166,167]. Despite these advances, substantial gaps remain. The tissue-specific regulation of IDO and TDO under stress conditions is not fully mapped, and their non-enzymatic roles in shaping the immune microenvironment remain underexplored [162,163,164]. Clinical trials with IDO inhibitors have yielded disappointing results, highlighting the need for dual IDO/TDO inhibition or combined targeting of downstream receptors [163,166,168]. Moreover, reliable biomarkers of enzyme activity and patient stratification strategies are lacking, while the dynamic regulation of heme incorporation and post-translational modifications introduces further complexity [161,162,163].

Elevated activity of the KYN pathway, particularly the accumulation of QA, has been strongly associated with depressive symptoms, impaired neurogenesis, and deficits in cognitive domains such as memory and executive function [169,170,171]. Neuroimaging and postmortem studies reveal that increased KYN metabolite ratios correlate with structural and functional brain abnormalities in depression. At the same time, animal models show that IDO-mediated pathway activation drives both neuroinflammation and cognitive decline [170,171,172]. Evidence also suggests that reduced KYNA, a putative neuroprotective metabolite, exacerbates cognitive impairment, especially in mood disorders [148,173,174]. These findings have stimulated interest in KYN-targeted therapies, ranging from small molecules to microbiota-based interventions [115,136,150,175,176]. Yet, significant questions remain unresolved. Central and peripheral measures of pathway activity often diverge, complicating biomarker development [148,174,177,178]. The causal links between specific metabolites and clinical symptoms require more mechanistic studies [124,169,172]. Translational efforts remain limited, with few robust clinical trials validating efficacy [148,175,177]. Furthermore, the role of gut microbiota in modulating KYN metabolism and cognitive outcomes is only beginning to be understood [136,179,180]. Longitudinal studies are essential for mapping disease trajectories and clarifying whether metabolite alterations differ across depression subtypes and cognitive phenotypes [148,172,177].

### 3.3. Gut–Brain Axis and Intestinal Microbiota: Peripheral Modulators of Tryptophan (Trp) Metabolism

**Figure 4:**
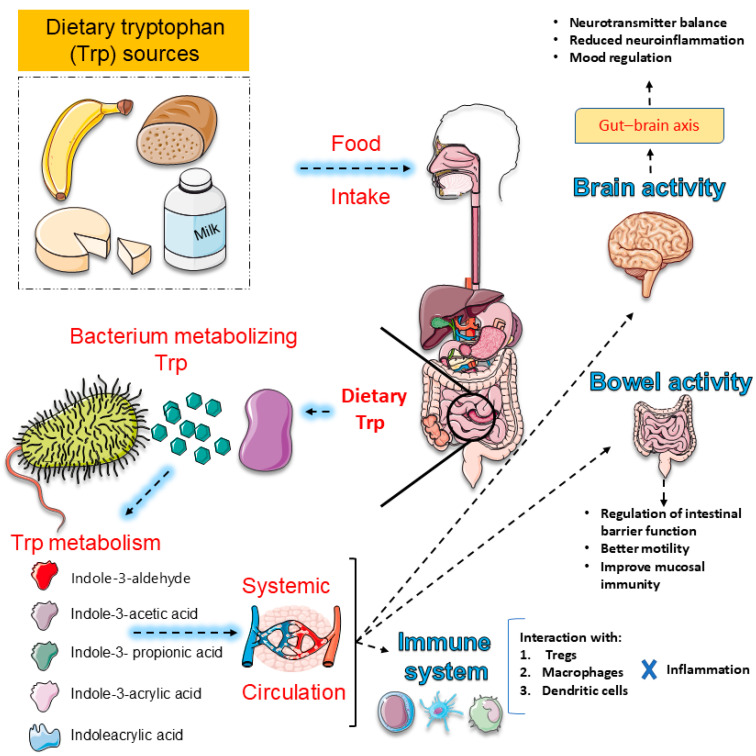
Dietary tryptophan (Trp) metabolism by gut microbiota and its systemic effects. Exogenous dietary Trp, derived from protein-rich foods, is metabolized by intestinal microbiota into indole derivatives, including indole-3-aldehyde (IAld), indole-3-acetic acid (IAA), indole-3-propionic acid (IPA), and indoleacrylic acid. These metabolites enter the systemic circulation and exert pleiotropic effects on host physiology. IAld activates the aryl hydrocarbon receptor (AhR), modulating mucosal immunity, while IPA provides neuroprotection through antioxidant activity. IAA regulates intestinal motility and epithelial function, whereas indoleacrylic acid strengthens gut barrier integrity. Collectively, these microbial metabolites influence brain activity (neurotransmission, neuroinflammation, mood), bowel function (motility, barrier homeostasis), and the immune system (immune tolerance, inflammation). This highlights the central role of gut microbiota–Trp metabolism in the gut–brain–immune axis.

The gut–brain axis functions as a bidirectional regulator of Trp metabolism linking the gastrointestinal tract with the central nervous system through neural, immune, endocrine, and metabolic pathways [181,182,183]. This system orchestrates critical processes that regulate neuroinflammation, energy metabolism, and psychiatric outcomes, with mounting evidence implicating gut microbiota and their metabolites as central mediators [160,184]. Dysbiosis has been consistently associated with depression, anxiety, schizophrenia, and neurodegenerative disorders, primarily through altered signaling across the vagus nerve, HPA axis, and immune-inflammatory cascades [185,186]. At the same time, microbial metabolites such as short-chain fatty acids and Trp derivatives modulate neurotransmission and glial function, further linking gut health to brain plasticity and cognition [181,187]. Yet, despite compelling evidence, substantial gaps remain. The precise molecular and cellular mechanisms underlying gut–brain interactions are incompletely defined, particularly regarding causal links [188,189]. Translation of probiotics, prebiotics, symbiotics, and FMT into reliable therapeutic strategies is hindered by inconsistent clinical results [183,188,190]. Biomarker development for diagnosis and treatment monitoring is still in its infancy, while most studies are restricted to narrow cohorts, limiting generalizability [183,188,191]. Longitudinal and mechanistic trials are urgently needed to clarify causality and optimize intervention strategies in psychiatric and neurodegenerative disorders [181,188,191]. Figure 4 summarizes the dietary Trp metabolism by gut microbiota and its systemic effects.

**Figure 5:**
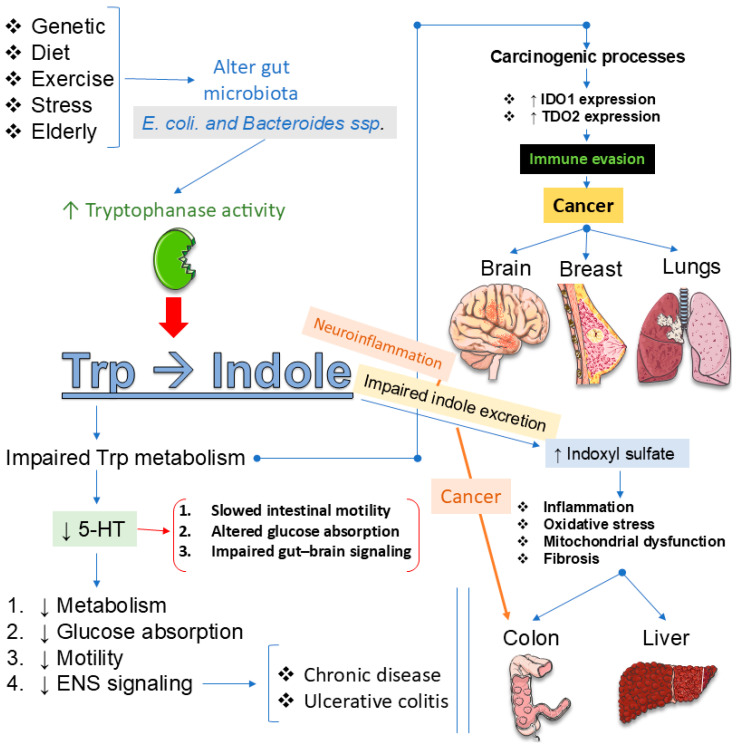
Tryptophan (Trp)–indole metabolism and its pathological consequences. Gut microbiota such as *Escherichia coli* and *Bacteroides* spp. metabolize dietary Trp via tryptophanase into indole. Host and environmental factors (genetics, diet, stress, aging, physical activity) modulate this pathway. Dysregulated Trp metabolism reduces serotonin (5-HT) availability, impairing enteric nervous system (ENS) signaling, metabolism, glucose absorption, and intestinal motility, contributing to chronic gastrointestinal disorders such as ulcerative colitis. Indole derivatives, particularly indoxyl sulfate (IS), accumulate when excretion is impaired, leading to systemic inflammation, oxidative stress, and cellular damage in the colon and liver. Overexpression of indoleamine 2,3-dioxygenase 1 (IDO1) and tryptophan 2,3-dioxygenase 2 (TDO2) further links altered Trp metabolism to carcinogenic processes in the brain, breast, and lungs. This highlights the central role of Trp–indole metabolism in the gut–brain–immune axis, cancer progression, and systemic disease.

As detailed earlier, microbial regulation of Trp availability shapes central signaling and mood through multiple converging pathways [136,160,192]. By modulating local inflammation, they alter the activity of enzymes that govern the diversion of Trp into the 5-HT and KYN branches, thereby influencing neurotransmission, neuroplasticity, and immune signaling [136,193,194]. Microbial metabolites also act directly: some species enhance 5-HT synthesis in the gut, while others promote KYN production or generate indole derivatives that engage the aryl hydrocarbon receptor to regulate neuroinflammation [195,196]. These interactions extend beyond the periphery, with microbial-driven metabolic shifts linked to depression, anxiety, and cognitive dysfunction in both preclinical models and clinical cohorts [136,160,192,194]. Yet, despite compelling evidence, essential uncertainties remain. The identity of key microbial species responsible for shaping Trp metabolism is incompletely defined, and the molecular mechanisms that orchestrate the balance between the 5-HT, KYN, and indole pathways remain unclear [194,197,198]. Translational progress is limited, as most findings derive from animal studies rather than large-scale human trials [194,199,200]. Therapeutic strategies such as probiotics, symbiotics, and dietary modulation hold promise, but their efficacy, safety, and mechanistic specificity need systematic evaluation [200,201,202]. Finally, reliable biomarkers to monitor microbiota-driven Trp metabolism and predict psychiatric outcomes are lacking, hindering the development of personalized interventions [194,197,203]. Figure 5 summarizes the Trp–indole metabolism and its pathological consequences.

**Figure 6:**
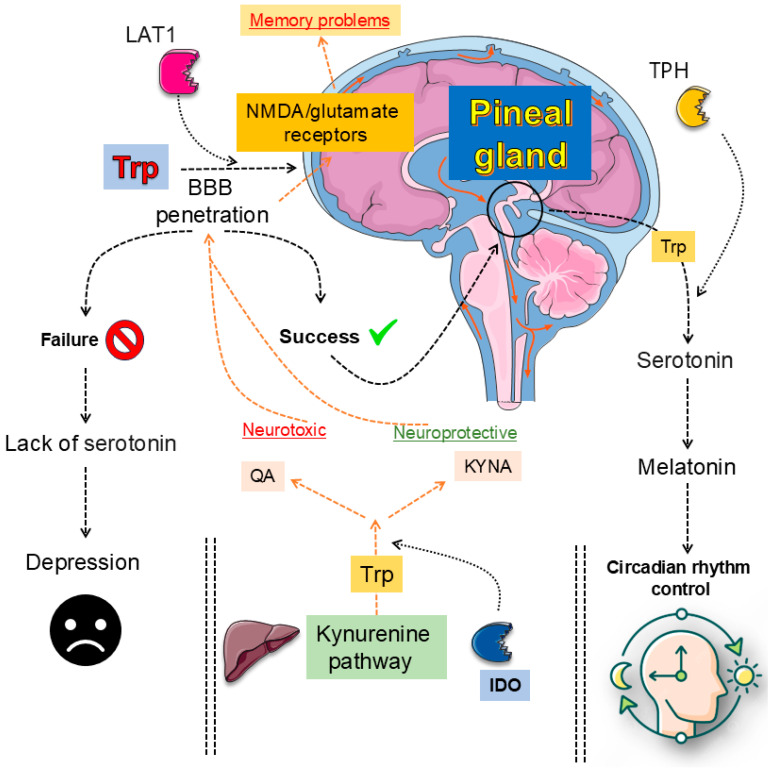
Dual pathways of tryptophan (Trp) metabolism and their neuropsychiatric implications. Trp crosses the blood–brain barrier (BBB) via LAT1 transporters and follows two competing metabolic routes. One route converts Trp to serotonin (5-HT) through tryptophan hydroxylase (TPH), which subsequently gives rise to melatonin, regulating circadian rhythms and mood. Impaired 5-HT synthesis contributes to depression and sleep disturbances. Alternatively, Trp is metabolized through the kynurenine (KYN) pathway, primarily via indoleamine 2,3-dioxygenase (IDO). This generates metabolites with opposing actions: quinolinic acid (QA), a neurotoxic N-Methyl-D-Aspartate (NMDA) receptor agonist that promotes excitotoxicity, oxidative stress, and cognitive dysfunction, and kynurenic acid (KYNA), a neuroprotective NMDA receptor antagonist that counterbalances QA’s toxicity. Dysregulation of the Trp–5-HT–KYN axis disrupts neurotransmission, neuroprotection, and circadian control, ultimately contributing to depression, memory problems, and neurodegeneration.

Peripheral metabolism does not operate in isolation. The gut microbiota strongly shapes the amount of Trp available for 5-HT synthesis or diversion into KYN metabolites, and these shifts can amplify or mitigate depressive risk. Emerging preclinical and clinical evidence suggests that targeting gut microbiota through probiotics, prebiotics, dietary phytochemicals, or even FMT can restore Trp balance, reduce neuroinflammation, and alleviate depressive symptoms [191,194,204]. Probiotics such as *Lactobacillus* and *Bifidobacterium* strains have been shown to enhance 5-HT availability, while prebiotics and phytochemicals promote beneficial microbial shifts that dampen KYN-driven neurotoxicity [205,206,207]. In animal models, microbiota modulation improves stress-induced depressive behaviors by redirecting Trp metabolism toward 5-HT rather than neurotoxic KYN derivatives, findings now echoed in early human trials reporting improved mood outcomes [205,206,208]. Nevertheless, significant gaps remain. Mechanistic clarity is limited, particularly regarding the molecular pathways by which specific microbial taxa regulate 5-HT and KYN branches [191,192,194]. Most clinical studies are small, short-term, and heterogeneous in design, making premature conclusions about efficacy and safety [208,209,210]. Inter-individual variability in microbiome composition complicates the development of standardized therapies, while long-term durability of benefits remains untested [191,194,209]. Finally, reliable microbial and metabolic biomarkers to guide patient stratification and therapeutic monitoring are lacking [191,194,209]. Addressing these gaps is essential for translating microbiota-based strategies into personalized, evidence-driven interventions for depression and related neuropsychiatric disorders. Figure 6 summarizes dual pathways of Trp metabolism and their neuropsychiatric implications.

## 4. Integrative Therapeutic Approaches as Modulators of Tryptophan (Trp) Metabolism with a Focus on Plant-Derived Dietary Strategies

Plant-derived compounds offer a versatile toolkit for modulating Trp metabolism at multiple biological levels, from enzyme activity to gut brain signaling [31,134,211]. Beyond simply providing additional Trp, plant foods and extracts combine substrate delivery with bioactive molecules that reshape the balance between 5-HT, KYN, and indole pathways. This dual action sets plant derived strategies apart from non-plant derived approaches such as synthetic supplements or pharmacological inhibitors that primarily target single receptors or enzymes. In this section, we outline how phytochemicals rebalance flux between serotonin and kynurenine pathways, influence sleep and cognition, and modify neuroinflammation, while also comparing clinical outcomes derived from plant based interventions with those reported for non-plant derived Trp or acute depletion paradigms [31,134,212]. By doing so, we position plant-derived Trp as a candidate for integrative depression management rather than as a simple nutritional adjunct. We integrate mechanistic insights with clinical observations, highlighting candidates such as 5-HTP, isoflavones, berberine, polyphenols, and Trp-rich dietary proteins [31,212,213]. Particular attention is given to bioavailability, pharmacokinetics, and microbiome interactions that shape therapeutic response [31,134,214]. By linking molecular targets to patient outcomes, this section frames an evidence-informed path toward translational strategies. It sets the stage for a critical appraisal of clinical trials in the subsections that follow.

### 4.1. Clinical Evidence for Plant-Derived Modulators of Tryptophan (Trp) Metabolism

Most available findings remain at an early stage. Many trials are small, short, or narrow in scope, so any mood or sleep improvements should be viewed as exploratory signals rather than evidence of clinical readiness [31,138,212]. Although several RCTs report changes in mood-related outcomes, these effects are usually modest, vary across studies, and remain unconfirmed in larger clinical samples [31,215,216]. Other interventions, such as Trp and magnesium-enriched Mediterranean diets tested in women with fibromyalgia, have explored the combined impact on sleep quality and anxiety. However, results remain mixed due to small cohorts and gender-restricted samples [217,218,219]. Broader inclusion criteria have encompassed patients with MDD or bulimia, while preclinical successes with compounds like albiflorin highlight the translational potential awaiting human validation [219,220,221]. Standard outcome measures include psychiatric rating scales, sleep quality indices, and neurochemical assays, allowing assessment of both symptomatic relief and mechanistic engagement [220,222,223]. 

The following table (Table 1) highlights relevant papers on dietary modulation of Trp in depression and related disorders, summarizing their focus, inclusion criteria, outcomes, and patient populations.

Randomized controlled trials investigating phytochemicals with modulatory effects on Trp metabolism have yielded encouraging, though heterogeneous, findings across domains of mood, anxiety, sleep, and cognition. Rosemary extract demonstrated significant reductions in anxiety and depressive symptoms among university students, along with measurable improvements in sleep quality and memory, suggesting a combined effect on mood regulation and cognitive processing [242,243,244]. Lavender oil (Silexan) consistently reduced generalized anxiety and restlessness while simultaneously enhancing sleep continuity, with benefits comparable to first-line anxiolytics but without sedative liabilities [245,246,247]. Trials with lemon balm further highlighted synergistic effects, showing improved psychological well-being, stress resilience, and sleep indices in adults with moderate emotional distress [245,248,249]. Beyond these botanicals, isoflavones and polyphenolic compounds have repeatedly been associated with improved affective stability and enhanced cognitive performance, likely mediated by their antioxidative and serotonergic interactions [250,251,252]. L-Trp supplementation and dietary enrichment strategies produced measurable anxiolytic and mood-stabilizing effects, although outcomes on sleep were inconsistent [251,252,253]. Berberine, while less frequently studied in formal RCTs, emerges from systematic analyses as a promising candidate due to its dual action on serotonergic pathways and neuroinflammation [15,252,254]. Taken together, the findings point to possible benefits across several symptom domains, yet the evidence remains preliminary because outcomes vary widely and most trials are too small to establish reliable clinical effects.

Clinical investigations of phytochemicals that influence Trp metabolism face several persistent limitations that limit their translational potential [255,256]. Variability in dosage and duration across trials complicates direct comparisons and limits the ability to establish optimal therapeutic regimens [255,257]. Intervention periods are often short, failing to capture long-term efficacy or safety profiles, particularly for compounds intended for chronic use in mood and anxiety disorders [255,257]. Patient populations are frequently homogeneous, with many studies confined to healthy adults or narrowly defined subgroups, reducing generalizability to clinically diverse cohorts [256,257]. Outcome measures further compound these issues, as trials employ a wide array of scales for mood, anxiety, and sleep, hindering reproducibility and meta-analytic synthesis [256,257]. Future research must prioritize methodological rigor by standardizing dosing protocols, harmonizing validated psychiatric and sleep measures, and extending trial durations [212,258]. Incorporating mechanistic endpoints through metabolomics, microbiome analysis, and neuroimaging would strengthen causal inference and bridge the translational gap [258,259]. 

### 4.2. Specific Tryptophan (Trp)-Rich Phytocompounds and Associated Clinical Outcomes

*Griffonia simplicifolia* seeds, a natural source of 5-HTP, have attracted considerable attention for their potential therapeutic effects on mood and anxiety disorders [260,261]. Small studies report improvements in mood, anxiety, and sleep, yet these observations remain preliminary because sample sizes are limited and replication is scarce [261,262]. These early signals are promising, but they cannot be interpreted as evidence of clinical readiness at this stage. These outcomes are biologically plausible, given that 5-HTP serves as the immediate precursor of 5-HT, bypassing the tightly regulated hydroxylation of Trp and directly fueling central serotonergic pathways [262,263]. Several narrative reviews underscore its promise in alleviating depression and insomnia, while preliminary trials report measurable gains in affective stability and reduced sleep latency [261,262]. Importantly, these findings align with mechanistic insights showing that oral 5-HTP elevates central 5-HT availability, thereby modulating emotional processing, stress resilience, and circadian regulation [262,264]. Although current evidence remains constrained by methodological limitations and the absence of large randomized controlled trials, the accumulating data suggest a clinically relevant role worth further exploration [238,265].

Soy isoflavones, particularly genistein and S-equol, have emerged as promising candidates for mood regulation through their intertwined effects on neuroinflammation and Trp metabolism [266,267]. Clinical and preclinical findings suggest that these phytochemicals suppress pro-inflammatory signaling cascades, including TLR4/NF-κB activation, while simultaneously shifting Trp utilization away from the KYN pathway and toward 5-HT synthesis [250,267]. This dual action is highly relevant for psychiatric disorders, as chronic inflammation and altered Trp metabolism often converge to lower 5-HT availability and exacerbate depressive and anxiety symptoms [130,268]. Trials in animal models demonstrate that soy isoflavones normalize 5-HT levels, reduce KYN accumulation, and enhance synaptic plasticity, culminating in reduced depressive-like and anxiety-related behaviors [250,267]. Although direct human clinical trials remain scarce, pilot studies in related contexts indicate improvements in affective stability, sleep quality, and cognitive performance [269,270]. Together, these findings position soy isoflavones as a biologically grounded strategy with significant translational potential awaiting rigorous clinical validation.

Berberine and albiflorin have recently emerged as two of the most compelling phytochemicals targeting the gut–brain axis, with converging evidence suggesting they can influence mood and cognition through microbiota-driven mechanisms [271,272]. Preclinical and early translational studies show that berberine exerts profound regulatory effects on gut microbial composition, enhancing the production of short-chain fatty acids and restoring gut barrier integrity, while simultaneously reducing peripheral and central inflammatory markers [271]. These changes translate into improved hippocampal 5-HT, dopamine, and BDNF signaling, resulting in alleviation of depressive-like behaviors and measurable gains in memory and learning in animal models of both depression and Alzheimer’s disease [273,274]. Albiflorin, a bioactive monoterpene glycoside, demonstrates a complementary mode of action: gut microbes metabolize it into benzoic acid, which penetrates the blood–brain barrier and inhibits D-amino acid oxidase, ultimately fostering neurogenesis and reducing depressive symptoms [275,276]. Although direct human clinical trials remain sparse, small-scale investigations and mechanistic reviews highlight reductions in depression severity, relief from anxiety, and preliminary cognitive benefits consistent with these gut–brain interactions [277,278]. Together, the data suggest that berberine and albiflorin represent promising candidates for modulating Trp metabolism, suppressing neuroinflammation, and enhancing cognitive resilience, warranting rigorous clinical validation to confirm their therapeutic potential [279,280].

Polyphenols and flavonoids have gained significant attention as multi-targeted modulators of brain health, with a growing body of evidence supporting their roles in mood stabilization, neuroprotection, and anti-inflammatory activity [281,282,283]. Clinical studies involving polyphenol-rich extracts demonstrate improvements in depressive and anxiety symptoms, often accompanied by reductions in circulating inflammatory cytokines such as TNF-α and IL-6, and improved systemic redox balance [253,284,285]. Flavonoids like naringenin and quercetin show particular promise, as they not only suppress pro-inflammatory pathways including NF-κB and MAPK but also promote hippocampal neurogenesis and synaptic plasticity, thereby enhancing resilience against stress-related pathology [252,283,286]. A notable mechanistic insight involves their potential modulation of the KYN pathway. By restraining the diversion of Trp toward neurotoxic KYN metabolites, polyphenols may preserve 5-HT synthesis while fostering neuroprotective KYNA balance [252,287,288]. Preclinical studies reinforce these effects, highlighting synergistic actions between flavonoids and gut microbiota in maintaining metabolic and neurotransmitter homeostasis [287,288,289]. While large-scale psychiatric trials remain limited, smaller investigations and indirect clinical evidence suggest tangible benefits in mood stabilization, cognitive function, and anxiety reduction [252,288,290]. Collectively, these findings underscore the translational potential of polyphenols and flavonoids as pleiotropic agents that target both immune and neurotransmitter pathways. Yet, they demand well-designed trials to establish their clinical efficacy in psychiatric populations.

**Figure 7:**
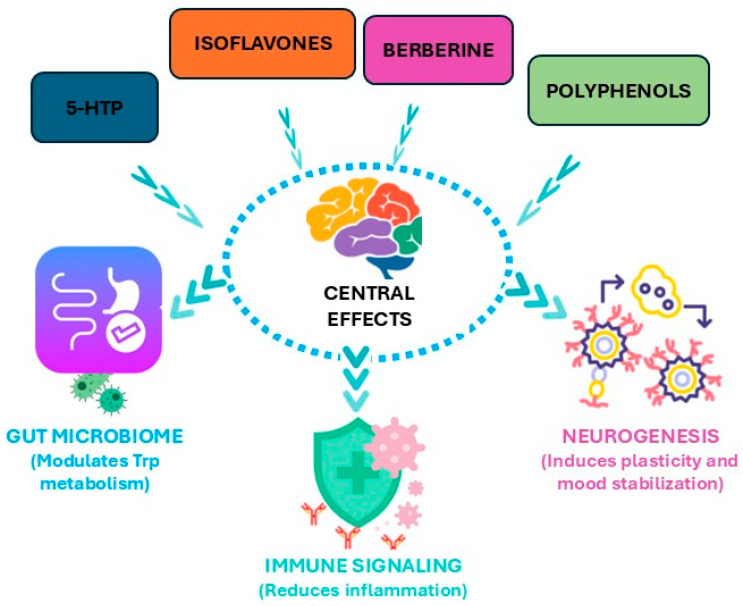
Mechanistic actions of phytochemicals in depression. Phytochemicals, including 5-hydroxytryptophan (5-HTP), isoflavones, berberine, and polyphenols, exert central effects through modulation of the gut microbiome (via tryptophan [Trp] metabolism), regulation of immune signaling (attenuation of inflammation), and stimulation of neurogenesis (enhancement of plasticity and mood stabilization), collectively contributing to antidepressant outcomes.

A visual figure illustrates the mechanisms by which key phytochemicals, such as 5-HTP, isoflavones, berberine, and polyphenols, influence depression biology (Figure 7). The diagram highlights their capacity to enhance 5-HT synthesis, regulate KYN metabolism, reduce neuroinflammation, and promote neurogenesis. Arrows connect peripheral processes, such as gut microbial modulation and immune signaling, to central effects in the brain, emphasizing the bidirectional nature of the gut–brain axis. By mapping these pathways together, the figure clarifies how dietary and phytotherapeutic interventions can complement pharmacological strategies. This visual summary strengthens understanding of mechanistic complexity while guiding translational applications in depression management.

## 5. Gaps and Controversies in Current Research

A central conceptual contribution of this review is the proposal that plant-derived Trp should be viewed as a systems level regulator of the Trp network rather than as a simple substitute for conventional supplementation [291,292]. By juxtaposing data from acute Trp depletion, non-plant derived supplementation, and plant-based interventions, we highlight that only the latter consistently engage three interconnected layers of biology, namely enzyme regulation along the KYN and 5-HT branches, microbiota dependent production of indoles, and immune modulation. This perspective moves beyond textbook descriptions of Trp pathways and invites hypothesis driven clinical trials that are explicitly designed to exploit these multi-level effects, for example by stratifying patients according to inflammatory status or microbiome composition when testing plant centered interventions. Research to date is marked by heterogeneity in trial design, inconsistency in dosing regimens, and insufficient mechanistic clarity, making it difficult to draw definitive conclusions about efficacy or safety [293,294]. Moreover, debates over bioavailability and the relative value of dietary versus supplemental sources remain unresolved [295]. This section critically examines the gaps and controversies that persist in the field, identifying key obstacles that must be addressed to advance evidence-based applications in psychiatry [292,295].

### 5.1. Identified Gaps in Current Research

Limited availability of long-term clinical trials on plant-derived Trp compounds represents a critical bottleneck in advancing depression therapeutics [296,297,298]. Most clinical investigations are confined to short durations, providing valuable insights into initial symptom reduction but leaving long-term efficacy and safety largely unexplored [32,298,299]. Historical work, such as Herrington’s comparative trial of L-Trp and electroconvulsive therapy, included a six-month follow-up but involved a limited sample, offering only fragmentary evidence regarding the durability of response [297]. This pattern highlights a broader issue: without longitudinal monitoring, it is impossible to determine whether early benefits represent genuine disease modification or transient relief [296,298,299]. Moreover, potential long-term risks, including serotonergic overstimulation, metabolic alterations, or shifts in gut–brain interactions, remain insufficiently characterized [12,298,300]. The absence of such data also obscures whether chronic administration leads to tolerance or adaptive physiological changes that diminish therapeutic value [297,298,300]. To honestly assess the role of plant-derived Trp compounds in the management of chronic depression, carefully designed, multi-year randomized controlled trials with repeated follow-up assessments are urgently required.

Inconsistency in dosing regimens, variability in formulation purity, and absence of standardized protocols represent significant obstacles to interpreting the therapeutic potential of plant-derived Trp compounds for depression treatment. Across clinical and preclinical studies, dosing practices vary widely, ranging from fixed low-dose supplementation to high-dose concentrated extracts, often administered at different frequencies. This lack of uniformity makes it nearly impossible to establish reliable dose–response relationships or determine optimal therapeutic windows. Differences in extraction methods, including solvent choice, temperature, and processing time, further affect yield, bioavailability, and pharmacological activity. Clinical trials using saffron or chamomile extracts, while reporting short-term improvements in depressive symptoms, often employ heterogeneous extraction techniques and undefined purity standards, preventing direct comparison or meta-analytic synthesis. Without harmonized dosing protocols and rigorous phytochemical standardization, conclusions regarding the safety and efficacy of these promising compounds remain provisional, limiting translation into clinical guidelines.

Although the Trp–KYN pathway has been extensively linked to depression and neuroinflammation, mechanistic clarity regarding how plant-derived phytochemicals act upon this axis is strikingly underdeveloped. Most studies remain descriptive, emphasizing correlations between altered Trp metabolism and psychiatric outcomes, while offering little insight into whether phytochemicals exert direct enzymatic modulation or act indirectly through immune regulation and microbiome interactions. For instance, trials investigating saffron and chamomile supplementation demonstrate short-term clinical benefits but fail to examine whether these effects are mediated by IDO inhibition, KYN–5-HT balance, or shifts in neuroinflammatory markers. Similarly, preclinical studies using Trp-rich diets in stress models suggest involvement of the gut–brain axis but lack detailed mapping of metabolite flux or cell-type-specific enzyme regulation. Without such mechanistic endpoints, it remains speculative whether phytochemical interventions primarily restore serotonergic tone, rebalance KYN derivatives, or attenuate inflammatory cascades. The paucity of molecular and biochemical investigations severely limits translation into targeted therapies. To overcome this, future research must integrate omics-driven profiling, metabolite quantification, and longitudinal immune assays within both animal and human trials. Only through such rigor can we disentangle direct versus indirect pathways and fully clarify how plant-derived Trp interventions modulate the biochemical underpinnings of chronic depression. Such uncertainties make it difficult to identify responders, to define therapeutic windows, or to design trials that align mechanistic action with clinical endpoints.

### 5.2. Controversies Regarding Clinical Efficacy and Bioavailability 

The debate surrounding dietary versus supplemental Trp remains unresolved, reflecting both biochemical complexity and methodological gaps in current research [138,301,302]. Opinions diverge sharply because supplements provide precision while dietary sources engage wider systemic pathways. Evidence supporting either scenario remains incomplete. Isolated supplementation offers clear pharmacological advantages, including predictable dosing, rapid absorption, and more consistent increases in serum Trp and downstream metabolites [301,303,304]. Systematic reviews demonstrate modest but reproducible improvements in mood and sleep quality with supplementation, yet optimal dosing strategies are far from established [301,303,304]. The evidence is encouraging but limited by small sample sizes, brief interventions, and inconsistent measurement panels. These constraints reduce confidence in effect size estimates and complicate comparisons across studies. In contrast, whole-food Trp intake introduces a range of variables: competition with other large neutral amino acids for transport across the blood–brain barrier, food matrix effects on absorption, and significant modulation by the gut microbiota [138,301,302]. Animal studies suggest that dietary Trp enhances intestinal barrier function and alters immune signaling pathways, implying potential systemic benefits distinct from supplementation, though mechanistic specificity remains elusive [305,306]. Although compelling, these findings primarily come from controlled laboratory settings and rarely translate directly into human physiology. Until head-to-head human trials are completed, conclusions remain tentative. For example, interventions in piglet and rodent models show that dietary Trp influences gut–brain and immune interactions more strongly than supplementation alone, but human trials directly comparing the two approaches are absent [307,308,309]. The resulting uncertainty complicates translation into clinical practice [195,308,309]. While supplements provide pharmacological precision, dietary sources may confer broader metabolic resilience, albeit with unpredictable bioavailability [309,310]. Head-to-head, long-term trials are urgently required to determine whether whole-food Trp and supplementation exert complementary or divergent therapeutic effects in depression [200,310,311,312].

Bioavailability remains one of the most contentious issues in evaluating dietary Trp and plant-based supplements, as absorption and metabolic fate are strongly shaped by gut microbiota composition, digestive efficiency, and individual genetic differences. While isolated supplementation produces predictable increases in plasma Trp, dietary sources are subject to competition with other large neutral amino acids and to modulation by the food matrix, resulting in less consistent outcomes. Compounding these pharmacokinetic challenges is the unresolved question of whether enhanced bioavailability directly translates into clinically meaningful improvements in depression, since mood regulation involves downstream serotonergic, KYN, and immune pathways. Finally, safety concerns linked to supplement impurities, as highlighted by the eosinophilia–myalgia syndrome outbreak, emphasize the critical need for rigorous pharmacokinetic and pharmacodynamic studies. Only with such evidence can bioavailability be meaningfully tied to therapeutic outcomes.

## 6. Clinical Translation: From Bench to Bedside

Research into plant-derived Trp compounds has expanded rapidly, yet critical uncertainties continue to limit their clinical translation [13,31,143]. Evidence from preclinical and early clinical studies highlights promising effects on mood, sleep, and stress resilience, but methodological gaps and unresolved controversies persist [31,240,301]. Questions surrounding long-term efficacy, optimal dosing, and mechanistic pathways remain central obstacles [138,143,303]. Likewise, debates over dietary versus supplemental sources and challenges of bioavailability further complicate interpretation [134,240,301]. This section examines these gaps and controversies, outlining unresolved issues that must be addressed before phytochemical modulation of Trp metabolism can be reliably integrated into psychiatric practice.

### 6.1. Practical Considerations for Clinical Application

Determining the effective and safe dosage of plant-derived Trp remains a central challenge in translating experimental findings into clinical practice [31,313,314]. Current evidence suggests a relatively wide therapeutic window, yet precise dosing guidelines are lacking, particularly for chronic depression [31,298,313]. Human studies indicate that daily intakes of 1–5 g are generally well tolerated, with a proposed NOAEL of 4.5 g/day in young adults [303,314,315]. These findings are consistent with short-term supplementation trials in healthy women, in which no safety concerns emerged after 3 weeks of use [298,304,315]. Animal studies further support this margin, showing no toxic effects at doses up to 2000 mg/kg/day over 90 days, underscoring a substantial buffer between practical and harmful exposure [31,240,316,317]. However, dosage requirements appear to vary depending on formulation: free L-Trp, fermented products, and protein-bound dietary forms display distinct bioavailability profiles that may influence clinical outcomes [31,304,316]. Patient-specific factors, such as age, sex, metabolic status, and comorbid conditions, are rarely addressed in current trials, leaving critical gaps in personalized dosing [31,304,316]. Significantly, historical episodes of eosinophilia–myalgia syndrome highlight the need for strict quality control, as impurities rather than Trp itself caused adverse events [240,298,318]. Collectively, available evidence supports cautious short-term use within established limits, while long-term efficacy and safety require rigorous randomized controlled trials across diverse populations.

Plant-derived Trp supplements are widely regarded as safe when used within recommended limits, yet their safety profile warrants careful consideration given both historical precedents and documented adverse reactions [314,319,320]. The most serious concern remains eosinophilia–myalgia syndrome, a rare but debilitating condition that emerged in the late 1980s due to contaminated L-Trp supplements [320,321,322]. Although later investigations confirmed that impurities rather than the amino acid itself were responsible, this episode underscores the importance of rigorous quality control and sourcing from reliable manufacturers [319,320,321]. This distinction is essential for current practice because modern formulations vary in quality, and safety remains dependent on manufacturing standards rather than the compound itself. Beyond this, clinical evidence indicates that most individuals tolerate doses up to 5 g per day for short durations without metabolic or systemic disturbances [314,318,323]. Nevertheless, higher intakes or prolonged use have occasionally been associated with nausea, tremor, dizziness, or excessive drowsiness. Interactions with serotonergic medications raise the additional risk of 5-HT syndrome, marked by neuromuscular rigidity, confusion, and fever, highlighting the need for medical oversight in polypharmacy contexts. Related compounds, such as 5-hydroxytryptophan, show similarly favorable safety profiles, though isolated case reports raise unresolved concerns that warrant continued vigilance [318,323,324]. Practical guidance emphasizes regular monitoring for muscle pain, skin changes, and gastrointestinal symptoms, patient education on recognizing side effects, and prompt discontinuation or dose reduction if adverse signs emerge, ensuring therapeutic benefit without undue risk [314,325,326].

Plant-derived Trp supplements raise important considerations regarding potential interactions with commonly prescribed medications, particularly antidepressants and anxiolytics that influence serotonergic signaling [31,134,327]. The most clinically significant risk is 5-HT syndrome. This potentially life-threatening condition can occur when Trp is combined with selective 5-HT reuptake inhibitors, 5-HT-norepinephrine reuptake inhibitors, monoamine oxidase inhibitors, or certain anxiolytics [31,134,327]. Characterized by agitation, hyperreflexia, autonomic instability, and, in severe cases, hyperthermia or coma, 5-HT syndrome underscores the need for clinicians to carefully review all concurrent serotonergic therapies before recommending Trp [142,328,329]. Beyond this, pharmacological overlap within the 5-HT and KYN pathways suggests that plant-derived Trp compounds could interfere with drugs targeting immune function or neuroinflammation, although robust clinical evidence remains sparse [31,134,330]. Pharmacokinetic interactions are less well defined but may involve competition for metabolic enzymes, potentially altering drug clearance and therapeutic effectiveness [142,329,331]. Contraindications include patients with a history of 5-HT syndrome, those taking multiple serotonergic agents, and individuals with unstable psychiatric or metabolic disorders [142,328,329]. Practical recommendations emphasize individualized assessment, use of the lowest effective dose, and structured patient education regarding early symptoms of adverse interactions [329,331,332]. Close monitoring and early discontinuation in the presence of concerning signs are essential to balance potential therapeutic benefits with safety in clinical application.

### 6.2. Personalized Medicine Approaches

Gut microbiota profiling is rapidly gaining attention as a tool for personalized depression management, with compelling evidence that microbial composition influences both Trp metabolism and therapeutic response to phytochemicals [192,194,333]. Altered microbiota profiles can shift the balance between 5-HT and KYN pathways, thereby modulating neuroinflammation and mood regulation [136,194,334]. Clinical studies now suggest that baseline microbiota signatures may predict antidepressant response: patients enriched in beneficial taxa, such as *Faecalibacterium prausnitzii* or *Bifidobacterium*, demonstrate higher remission rates, while reduced diversity often correlates with treatment resistance [333,335,336]. Early trials of Trp-rich dietary interventions and probiotics also reveal that therapeutic efficacy may hinge on an individual’s microbial ecosystem, with some patients showing robust mood improvement and others little benefit despite identical regimens [333,335,337]. This variability highlights the potential of stool-based profiling to guide personalized strategies, matching patients to dietary, probiotic, or phytotherapeutic interventions most likely to be effective [208,335,338]. Emerging data also point to machine learning approaches that integrate microbial, metabolic, and cytokine biomarkers for predictive modeling of treatment outcomes [338,339,340]. Yet translation into practice is still nascent: large-scale, longitudinal trials and functional multi-omics studies are essential to confirm which microbial profiles drive therapeutic benefit [115,194,336]. Ultimately, microbiota-informed stratification could transform the management of depression into a more precise and individualized discipline.

Genetic polymorphisms in key enzymes regulating Trp metabolism, such as IDOs, TDO, and TPH, play a decisive role in shaping vulnerability to depression and responsiveness to therapeutic interventions [192,194,333]. Variants in these genes influence the metabolic fate of Trp, determining whether it is directed toward 5-HT synthesis or shunted into the KYN pathway, with downstream consequences for neuroinflammation and mood regulation [115,136,334]. Clinical data suggest that polymorphisms in TPH2 and 5-HT transporter-linked regions correlate with depression severity and treatment resistance. At the same time, IDO and IFN-γ gene variants are associated with enhanced KYN production, amplifying immune-driven depressive symptoms [113,341,342]. Postpartum depression studies also reveal IDO-related polymorphisms as predictors of higher risk, underscoring the clinical relevance of pathway-specific genetic variation [341,343,344]. These insights highlight the potential of gene profiling to guide individualized treatment, in which patients with high-risk alleles may benefit more from interventions targeting the KYN pathway or from plant-derived Trp formulations designed to enhance serotonergic flux [10,113,341]. Although current evidence remains fragmented, integrating genotyping into personalized medicine frameworks could enable patient stratification, prediction of treatment outcomes, and rational selection of phytotherapeutic strategies [341,344,345]. Expanding multi-ethnic genetic studies and intervention trials is essential to validate these applications and move toward precision psychiatry [346].

## 7. Future Perspectives and Research Directions

Clinical translation represents the critical step of transforming mechanistic insights and preclinical discoveries into therapeutic strategies that can be safely and effectively applied in patient care [150,347,348]. For plant-derived Trp and related compounds, this bridge from bench to bedside demands careful attention to dosing, safety, interactions, and long-term monitoring [143,349,350]. Equally important is the integration of personalized medicine approaches, in which genetic, metabolic, and microbiome profiles guide therapeutic decisions [351,352,353]. By addressing these considerations, clinical application moves beyond experimental promise to evidence-based strategies capable of shaping future psychiatric treatments.

### 7.1. Advancing Clinical Evidence Through Robust Study Designs

Large-scale, multi-center clinical trials represent the critical next step for validating plant-derived Trp therapies in depression [10,30,354]. While early-phase studies and small-scale trials suggest therapeutic promise, their limited sample sizes, short durations, and restricted demographic scope constrain clinical interpretation [115,354,355]. Multi-center designs allow for the inclusion of diverse populations across ethnic, geographic, and socioeconomic backgrounds, thereby addressing genetic polymorphisms in enzymes such as IDO, TDO, and TPH that influence Trp metabolism and treatment responsiveness [10,30,355]. Such diversity is essential to move beyond narrow, context-specific findings and toward results with broad clinical applicability. Equally important, multi-center collaborations enhance statistical power, enabling the detection of subtle treatment effects and the evaluation of subgroup responses [356,357]. Coordinated protocols across research networks also promote methodological consistency, ensuring that differences in formulation, dosing, and biomarker assessments do not confound outcomes [358,359,360]. Promising applications include testing whether specific genotypic or microbiota-defined subgroups derive greater benefit from phytotherapeutic interventions, a question that can be adequately addressed only through large, well-controlled trials [361,362,363]. Beyond efficacy, safety monitoring on a global scale provides crucial insight into rare adverse events and long-term tolerability [359,360,363]. Ultimately, multi-center trials create the foundation for standardized guidelines, ensuring that plant-derived Trp interventions are evaluated with the rigor required for integration into precision psychiatry [364,365,366,367,368].

Standardization of methodologies and outcome measures is indispensable for advancing plant-derived Trp therapies from promising pilot studies to clinically actionable interventions [369,370,371]. At present, heterogeneity in dosing protocols, extraction techniques, biomarker panels, and outcome assessments undermines comparability across trials and limits the ability to generate robust meta-analyses [372,373]. For instance, differences in extraction solvents and processing conditions can yield markedly divergent metabolite profiles, complicating interpretation of therapeutic efficacy [242,372]. Similarly, clinical studies often apply variable dosing schedules—ranging from milligram-scale supplementation to multi-gram intakes—without harmonized reference points, making it difficult to establish optimal therapeutic windows [374,375]. Biomarker selection presents another challenge: while some studies emphasize 5-HT and KYN metabolites, others focus on inflammatory cytokines or gut microbiota-derived indoles, creating fragmented datasets that resist synthesis [376,377,378]. Outcome assessment tools are equally inconsistent, with trials variously employing clinician-rated depression scales, patient self-reports, or biochemical endpoints [375,378]. A shift toward standardized protocols—including consensus dosing frameworks, validated extraction procedures, and a unified “indolome” biomarker panel—would significantly enhance reproducibility and clinical interpretability [379,380,381]. Such consistency is particularly critical for large-scale, multi-center trials, where methodological alignment ensures that findings are both statistically reliable and generalizable [358,379,382]. Ultimately, methodological standardization is not a technical detail but the foundation for transforming plant-derived Trp research into credible, guideline-ready therapies.

Long-term follow-up studies are indispensable for establishing the actual clinical value of plant-derived Trp therapies in depression management [10,31,383]. While short-term trials consistently report symptomatic improvements, they cannot determine whether these benefits persist over months or years, nor can they capture delayed adverse effects that may only emerge with prolonged use [297,298,383]. Longitudinal research is therefore essential to assess the durability of mood stabilization, sustained neurobiological changes, and overall psychiatric outcomes [10,32,384]. Equally important is evaluating patient adherence, as real-world compliance often declines over time and directly influences therapeutic success [32,298,385]. Early evidence from nutritional and probiotic interventions suggests that plant-derived Trp compounds can modulate 5-HT and KYN pathways in ways that might yield lasting benefit, yet without systematic long-term monitoring, such effects remain speculative [12,299,386]. Furthermore, the safety profile of chronic use is not fully characterized, particularly in populations with comorbidities or concurrent pharmacotherapies [241,383,384]. Long-term follow-up would also clarify whether plant-derived formulations maintain efficacy or whether tolerance, metabolic adaptation, or microbiota shifts reduce their impact over time [31,383,387]. Only through rigorous, multi-year studies with repeated biochemical, clinical, and functional assessments can the field move from preliminary promise to evidence-based recommendations that ensure both efficacy and safety in chronic depression care.

### 7.2. Combined Pharmacological and Dietary Intervention Strategies

Integrating dietary interventions with standard pharmacological treatments offers a compelling strategy for enhancing depression management [388,389,390]. Evidence from randomized controlled trials, such as the SMILES study, demonstrates that improving diet alongside usual care results in significantly greater reductions in depressive symptoms and higher remission rates than control conditions [390,391,392]. Meta-analyses further support these findings, showing that dietary modifications consistently improve mood outcomes, even when participants are already receiving antidepressants [393,394,395,396]. Nutrient-focused approaches, including supplementation with omega-3 fatty acids, vitamin D, and probiotics, have shown additive benefits, with effects on neurotransmitter synthesis, immune regulation, and gut–brain communication that complement pharmacological mechanisms [388,396,397]. Together, these findings highlight the rationale for combined strategies that address both biological and lifestyle determinants of mental health.

The integration of diet with pharmacotherapy also has practical advantages. Nutritional interventions may mitigate side effects commonly associated with antidepressant use, such as weight gain, fatigue, and gastrointestinal disturbances, thereby improving tolerability and adherence [396,397,398]. Patients often perceive dietary changes as more holistic and less stigmatizing than medication alone, thereby enhancing engagement and long-term treatment compliance [399,400,401]. From a mechanistic perspective, dietary modulation of inflammation, oxidative stress, and Trp metabolism provides synergistic support to monoamine-based drug action [389,394,402]. Thus, combining dietary and pharmacological approaches is not merely additive but can produce synergistic effects, optimizing both symptom relief and overall health outcomes [389,403]. This integrated model represents one of the most promising directions for personalized, sustainable depression care.

Future research on combined dietary Trp and antidepressant interventions requires rigorously designed clinical trials that address both short- and long-term outcomes. The most promising model is a randomized, double-blind, placebo-controlled design with multiple parallel arms: antidepressant plus Trp, antidepressant plus placebo, Trp alone, and full placebo [296,385,404]. Such a structure allows investigators to disentangle additive and synergistic effects while also clarifying the standalone impact of dietary supplementation [32,385,404]. Control conditions should be carefully balanced to avoid expectancy bias, and stratification by baseline nutritional status or genetic polymorphisms in Trp metabolism (e.g., IDO, TDO, TPH variants) would enhance precision in interpreting outcomes [32,210,405].

Biomarker monitoring should be a central feature of these trials. Serial assessments of plasma Trp, KYN metabolites, 5-HT levels, inflammatory cytokines, and sleep parameters would help map mechanistic pathways linking supplementation to clinical response [406,407,408]. Alongside these biological endpoints, standardized psychiatric outcome measures—such as HDRS and BDI scores—should be collected, complemented by cognitive and emotional processing tasks to capture subtle shifts in mood regulation [409,410,411]. Adherence can be monitored with digital tracking tools and serum metabolite checks [412,413,414]. A duration of at least 8–12 weeks is needed to capture the short-term efficacy, but follow-up at 6 to 12 months will be critical to assess durability, safety, and adherence in real-world contexts [415,416]. These models would not only clarify the therapeutic potential of combined interventions but also provide insight into patient subgroups most likely to benefit. By integrating dietary, pharmacological, and mechanistic perspectives, such studies could build the evidence base needed to translate plant-derived Trp strategies into precision-guided clinical practice.

### 7.3. Leveraging Biotechnological Innovations for Personalized Depression Management

Precision nutrition is redefining how dietary strategies are applied in depression care by emphasizing individualized interventions informed by genetic and metabolic profiling [417,418,419]. Nutritional genomics has revealed that polymorphisms in genes such as SLC6A4 and BDNF can influence how individuals respond to nutrient-based therapies, including plant-derived Trp [417,420,421]. These differences suggest that personalized dosing algorithms may eventually replace one-size-fits-all approaches, although such models remain experimental at present. For example, specific 5-HT transporter variants may determine whether Trp is preferentially metabolized into 5-HT or diverted into the KYN pathway, directly affecting mood outcomes [332,422,423]. Integrating this genetic information into clinical planning could enable targeted dietary prescriptions tailored to a patient’s unique metabolic capacity, thereby improving the precision and efficacy of treatment.

Beyond genomics, advanced biomarker analyses provide powerful tools to individualize dietary interventions [424,425,426]. Gut microbiota profiling typically involves sequencing approaches that characterize intestinal microbial composition and functional metabolic capacity. Profiling gut microbiota, serum metabolites, and inflammatory markers allows clinicians to predict and monitor how patients metabolize Trp-rich foods or supplements [427,428,429]. Emerging evidence shows that personalized diets can reduce depressive symptoms, alter microbial composition, and improve quality of life in both older adults and adolescents [393,399,427]. These findings suggest that combining biomarker-driven monitoring with dietary tailoring may optimize long-term outcomes [425,426,430]. Importantly, precision nutrition also considers comorbidities: in patients with obesity or diabetes, individualized nutritional plans have demonstrated benefits not only for metabolic regulation but also for mood stabilization [265,428,430]. Together, these insights support a move away from generic dietary recommendations toward integrative, personalized protocols. When fully developed, such strategies could complement pharmacological treatments and establish plant-based Trp interventions as a cornerstone of personalized psychiatry.

Microbiome-targeted therapeutic approaches are emerging as innovative strategies for addressing depression by modulating gut–brain communication and regulating Trp metabolism [383,431,432]. Probiotics and prebiotics are the most extensively studied, with randomized controlled trials showing that probiotic supplementation can alleviate depressive symptoms, maintain microbial diversity, and enhance serotonergic signaling [386,433,434]. Specific strains, such as *Lactobacillus* and *Bifidobacterium*, have been linked to improved mood outcomes and altered brain activation patterns, suggesting that targeted microbial interventions may complement conventional pharmacotherapy [337,435,436]. Prebiotics, by selectively stimulating beneficial gut bacteria, also influence the production of neuroactive metabolites, offering an indirect yet consequential means of modulating the Trp–KYN pathway [31,431,437].

FMT represents a more radical but promising intervention [204,438,439]. Preclinical studies in rodent models demonstrate that transplantation from healthy donors reduces depressive behaviors, restores neurotransmitter balance, and dampens neuroinflammatory processes [440,441,442]. Early clinical findings echo these benefits, although long-term safety and efficacy remain insufficiently studied [443,444,445]. Beyond these approaches, engineered microbial therapies are on the horizon, with genetically modified strains designed to produce specific neuroactive compounds or regulate metabolic flux through Trp-related pathways [194,200,333]. When combined with multi-omics profiling, these advanced strategies could allow for precision-guided microbial interventions tailored to an individual’s baseline microbiome and metabolic profile [194,204,333]. Collectively, probiotics, prebiotics, FMT, and engineered microbes hold strong potential to enhance therapeutic outcomes, particularly when integrated into holistic models of depression care that also consider diet, pharmacology, and genetics.

## 8. Conclusions

This review synthesizes current knowledge on plant-derived Trp interventions and outlines how these mechanisms intersect with psychiatric research. The evidence highlights potential applications while clarifying where further work is needed. By weaving together evidence from neuroinflammation, KYN metabolism, gut–brain interactions, and phytochemical modulation, the article provides a comprehensive framework that highlights both theoretical insights and translational promise. Notably, the findings demonstrate that phytocompounds can recalibrate serotonergic balance, temper neurotoxic cascades, and enhance resilience through multimodal pathways, supporting their integration alongside conventional antidepressants. At the same time, methodological limitations—heterogeneous dosing protocols, brief intervention periods, and insufficient long-term safety data—demand carefully designed clinical trials. Future research should prioritize precision nutrition strategies, biomarker-guided interventions, and longitudinal monitoring to elucidate the sustained impact of these therapies fully. Advancing this field will require interdisciplinary collaboration and coordinated multi-center trials that reflect real-world diversity and consistent methodological standards. The promise is real, yet current data remain preliminary because most trials are underpowered or methodologically heterogeneous. Larger, more mechanistically grounded studies are needed to confirm clinical relevance and advance the field toward safer, more effective, and biologically informed treatment paradigms. A significant gap remains in the absence of coordinated multi-center RCTs that apply harmonized dosing frameworks, standardized extraction procedures, and unified biomarker panels. Without such designs, it is difficult to determine whether early signals of efficacy translate into durable clinical benefit.

## Figures and Tables

**Table 1:** Summary of studies investigating dietary interventions for Trp modulation in depression and related disorders and conditions. The table outlines study design, patient populations, interventions, primary outcomes, and key observations.

Recovered/Remitted Depression				
**Study Design and Population**	**Intervention/Control**	**Main Outcomes**	**Key Notes**	**Ref.**
Crossover, DB, 23 recovered depressed vs. 20 healthy controls (NL)	α-lactalbumin (Trp-rich) vs. casein	Improved memory; impaired simple motor performance	No effect on mood	[224]
RCT, DB, crossover, 16 recovered-depressed subjects vs. 17 healthy subjects (UK and NZ)	ATD vs. placebo	No mood change overall	ATD decreased MMSE parameters in the recovered-depressed group	[225]
**Patients with Depressive Symptoms/Depression**				
**Study Design and Population**	**Intervention/Control**	**Main Outcomes**	**Key Notes**	**Ref.**
RCT, crossover, 30 subjects with MDD (MY)	Talbinah (Trp-rich barley) vs. control	↓ depression and stress (DASS, POMS)	Multidomain improvement	[226]
RCT, DB, 60 subjects with MDD (IN)	5-HTP vs. fluoxetine (8 wks)	Both ↓ HDRS (by wk 2)	Comparable efficacy	[227]
RCT, DB, 25 patients with Parkinson’s disease and depressive/apathy symptoms (IT)	5-HTP (50 mg) vs. placebo (crossover: 4 wks + 4-wk washout + 4 wks)	↓ depression (BDI-II, HDRS)	5-HTP improved depressive symptoms but not apathy in Parkinson’s disease	[228]
**High-Risk/Vulnerable Groups**				
**Study Design and Population**	**Intervention/Control**	**Main Outcomes**	**Key Notes**	**Ref.**
Placebo-controlled, DB, 29 high stress-vulnerable vs. 29 low stress-invulnerable adults (NL)	α-lactalbumin (Trp-rich) vs. casein	Trp improved mood (POMS) and ↑ Trp/LNAA ratio in the stress-vulnerable group, ↓ in cortisol in both groups	Antidepressant-like effect under stress	[229]
RCT, DB, 20 high vs. 18 low cognitive reactivity subjects (NL)	Trp-rich hydrolysate vs. casein	Trp improved positive mood, and ↓ cortisol after stress	Both groups benefited physiologically	[230]
Placebo-controlled, crossover, DB, 20 high-risk (family history) vs. 19 low-risk (CA)	ATD vs. control	High-risk: lowering mood (POMS)	ATD amplified vulnerability markers	[231]
**Bulimia**				
**Study Design and Population**	**Intervention/Control**	**Main Outcomes**	**Key Notes**	**Ref.**
RCT, DB, 26 unmedicated women with bulimia vs. 13 women with bulimia treated with SSRIs vs. 25 healthy control women (CA)	ATD vs. control (balanced amino acids)	ATD ↓ mood in all; ↑ urge to binge eat in bulimia + SSRIs	5-HT depletion linked to bulimic relapse, especially with SSRI use	[232]
**Healthy Volunteers**				
**Study Design and Population**	**Intervention/Control**	**Main Outcomes**	**Key Notes**	**Ref.**
RCT, DB, 38 healthy adults (UK)	Trp (1 g, 3x/day, 14 d) vs. placebo	Trp increased recognition of happy faces, reduced recognition of disgusted expressions (females)	Positive emotional bias in the processing of emotional material with stronger effects in women	[233]
RCT, DB, 20 healthy adults (CA, NL, and FR)	Trp (3 g/day, 3 wks) vs. placebo (crossover with a 2-week washout period)	↑ plasma/urine KYN and indoles; no effect on mood and no GI symptoms	Trp triggered immunomodulatory metabolic pathways	[234]
RCT, DB, 47 healthy adults (NL)	Trp (2.8 g/day, 6 d) vs. placebo	↑ rejection of unfair offers	Prolonged Trp may affect social decision-making	[235]
RCT, DB, 18 healthy adults (NL)	Trp-rich hydrolyzed protein vs. α-lactalbumin (Trp-rich) vs. casein vs. pure Trp vs. Trp-containing synthetic peptide	Hydrolyzed Trp and pure Trp improved mood (acute); hydrolyzed Trp caused an increase in plasma Trp/LNAA faster and greater than α-lactalbumin	Hydrolyzed Trp is more efficient in increasing brain Trp and 5-HT function compared with α-lactalbumin and pure Trp	[236]
**Elderly**				
**Study Design and Population**	**Intervention/Control**	**Main Outcomes**	**Key Notes**	**Ref.**
Comparative study, SB, 35 elderly subjects (ES)	Trp-enriched cereals vs. standard cereals vs. usual diet	↑ sleep quality, ↓ anxiety and depression	Improved age alterations on the sleep/wake cycle	[237]
RCT, DB, 25 elderly individuals with mild cognitive impairment (IT)	DHA-phospholipids + melatonin + Trp vs. placebo, 12 weeks	↑ cognitive function, ↑ nutrition, ↑ olfactory sensitivity	MMSE improved, positive trend for verbal fluency	[238]
RCT, DB, 14 elderly subjects (NL)	Trp + nicotinic acid + nicotinamide vs. control, 32 days	No effect on mitochondrial or muscle function	↑ NAD+ metabolism, no functional benefit	[239]
Open-label clinical trial, 40 elderly individuals with depression/sleep disorders (PL)	Trp-rich diet (25 mg/kg/d), 12 weeks	↓ depression and insomnia scores, improved Trp metabolism	ISI and HDRS scores halved post-intervention	[240]
**Fibromyalgia**				
**Study Design and Population**	**Intervention/Control**	**Main Outcomes**	**Key Notes**	**Ref.**
RCT, 22 women with fibromyalgia (ES)	Mediterranean diet enriched with high-dose Trp (60 mg) and Mg (60 mg) vs. standard Mediterranean diet	↓ anxiety, ↓ mood disturbance, ↓ eating disorder symptoms, ↓ body image dissatisfaction; no improvement in sleep quality	Significant improvements in psychological variables	[217]
**Large-Scale Observational**				
**Study Design and Population**	**Intervention/Control**	**Main Outcomes**	**Key Notes**	**Ref.**
Clinical trial, 29,687 adults (US) (NHANES 2001–2012)	Mean Trp intake: 826 mg/d (observational, no intervention)	Higher Trp intake associated with ↓ depression severity (PHQ-9), ↑ sleep duration	No adverse effects on liver, kidney, or metabolism	[241]

5-HT, serotonin; 5-HTP, 5-Hydroxytryptophan; ↑, Increase; ↓, Decrease; ATD, Acute Tryptophan Depletion; BDI-II, Beck Depression Inventory-II; CA, Canada; DASS, Depression Anxiety Stress Scales; DB, Double-Blind; DHA, Docosahexaenoic Acid; ES, Spain; FR, France; GI, Gastrointestinal; HDRS, Hamilton Depression Rating Scale; IN, India; ISI, Insomnia Severity Index; IT, Italy; KYN, Kynurenine; LNAA, Large Neutral Amino Acids; Mg, Magnesium; MDD, Major Depressive Disorder; MMSE, Mini-Mental State Examination; MY, Malaysia; NL, Netherlands; NZ, New Zealand; PHQ, Patient Health Questionnaire-9; PL, Poland; POMS, Profile of Mood States; RCT, Randomized Controlled Trial; SB, Simple-Blind; SSRIs, Selective Serotonin Reuptake Inhibitors; Trp, Tryptophan; UK, United Kingdom; US, United States.

## Data Availability

No new data were created or analyzed in this study. Data sharing is not applicable to this article.

## Abbreviations

The following abbreviations are used in this manuscript:

3-HK	3-hydroxykynurenine
5-HT	serotonin
5-HTP	5-hydroxytryptophan
AhR	aryl hydrocarbon receptor
AMPA	α-amino-3-hydroxy-5-methyl-4-isoxazolepropionic acid
BDI	Beck Depression Inventory
BDNF	brain-derived neurotrophic factor
CDK5	cyclin-dependent kinase 5
FMT	fecal microbiota transplantation
HDRS	Hamilton Depression Rating Scale
HPA	hypothalamic–pituitary–adrenal
IDO	indoleamine 2,3-dioxygenase
IFN	interferon
IL	interleukin
KAT	kynurenine aminotransferases
KMO	kynurenine 3-monooxygenase
KYN	kynurenine
KYNA	kynurenic acid
MAPK	mitogen-activated protein kinase
MDD	major depressive disorder
NF-κB	nuclear factor kappa B
NLRP3	NLR family pyrin domain-containing protein 3
NMDA	N-Methyl-D-Aspartate
NOAEL	No Observed Adverse Effect Level
QA	quinolinic acid
QPRT	quinolinate phosphoribosyltransferase
RCT	randomized controlled trial
SSRI	selective serotonin reuptake inhibitor
TDO	tryptophan 2,3-dioxygenase
TLR4	toll-like receptor 4
TNF-α	tumor necrosis factor-alpha
Trp	tryptophan
TPH	tryptophan hydroxylase
VEGF	vascular endothelial growth factor
